# The mechanism and efficacy of GLP-1 receptor agonists in the treatment of Alzheimer’s disease

**DOI:** 10.3389/fendo.2022.1033479

**Published:** 2022-11-17

**Authors:** Haiyang Du, Xiaoyu Meng, Yu Yao, Jun Xu

**Affiliations:** ^1^ Division of Orthopedics, Department of Orthopedics, The Second Affiliated Hospital of Harbin Medical University, Harbin, China; ^2^ Division of Endocrinology, Department of Internal Medicine, Tongji Hospital, Tongji Medical College, Huazhong University of Science and Technology, Wuhan, China; ^3^ Branch of National Clinical Research Center for Metabolic Diseases, Hubei, China

**Keywords:** Alzheimer’s disease, GLP-1R agonists, cognitive function, amyloid beta, tau phosphorylation

## Abstract

Since type 2 diabetes mellitus (T2DM) is a risk factor for Alzheimer’s disease (AD) and both have the same pathogenesis (e.g., insulin resistance), drugs used to treat T2DM have been gradually found to reduce the progression of AD in AD models. Of these drugs, glucagon-like peptide 1 receptor (GLP-1R) agonists are more effective and have fewer side effects. GLP-1R agonists have reducing neuroinflammation and oxidative stress, neurotrophic effects, decreasing Aβ deposition and tau hyperphosphorylation in AD models, which may be a potential drug for the treatment of AD. However, this needs to be verified by further clinical trials. This study aims to summarize the current information on the mechanisms and effects of GLP-1R agonists in AD.

## Introduction

Alzheimer’s disease (AD), a global public health priority, is the most common neurodegenerative disease (1). AD is recognized as the leading cause of disability and death, and the progressive cognitive dysfunction in AD patients seriously affects the quality of life (2). According to previous research, there were 46.8 million people suffering from dementia worldwide in 2015 and AD is identified as the leading cause for dementia (3). Due to the severe cognitive impairment of AD patients, their treatment and care require substantial economic and financial support, causing serious damage to global economic development (3). The main pathological features of AD are amyloid plaques and neurofibrillary tangles (NFTs) as well as neuroinflammation and oxidative stress in the brain (2, 4, 5). Many studies have revealed that diabetes, insulin resistance and aging are major risk factors for AD (6–9). Interestingly, previous studies have shown brain insulin resistance in AD patients (10, 11). Therefore, AD is also called “type 3 diabetes” (12, 13). Unfortunately, so far, there is no effective treatment for AD.

Some human and animal studies show that insulin can accelerate the clearance level of Aβ in the brain and affects the phosphorylation of tau protein, meanwhile, enhances the synaptic activity and plasticity of neurons (14–16). It produces beneficial effects if administered for short periods, because raising peripheral insulin levels acutely increases insulin levels in the brain and cerebrospinal fluid (CSF) (17–19). However, sustained high levels of circulating insulin may conversely exert a negative influence on cognitive function, due to prolonged peripheral hyperinsulinemia down-regulates insulin receptors at the blood-brain barrier (BBB) and reduces insulin transport into the brain, leading to brain insulin resistance (BIR) (17–19).

Glucagon-like peptides 1 (GLP-1), derived from intestinal L cells, is an incretin hormone. GLP-1 is a target for treatment of diabetes because of the primary peripheral functions of inducing insulin secretion from pancreatic β cells, gut emptying and inhibiting glucagon secretion which results in lower blood glucose levels (20). Continuous administration of natural GLP-1 increases insulin levels and results in lower blood glucose and hemoglobin A1C (HbA1c) levels in patients with type 2 diabetes mellitus (T2DM) (20). GLP-1 has a short half-life of only two minutes, due to renal clearance and endogenous GLP-1 is degraded by the enzyme dipeptidyl peptidase IV (DPP-4) (20–22). For these reasons, GLP-1 analogues and DPP-4 inhibitors were synthesized to prolong the half-life of GLP-1.

GLP-1 can also be produced by neurons in central nervous system (CNS) (20, 23). GLP-1 receptors (GLP-1R) expression has been detected throughout the CNS including the hippocampus, neocortex, hypothalamus, and cerebellum (17, 20, 24). GLP-1R are expressed by neurons, glia cells do not express this receptor but induced expression when activated in an inflammatory response (17, 25). Recently, GLP-1 and its agonists have been found to have good neuro-regulation and protection effects in animal models (26–28). Therefore, this review summarizes the current information on the mechanisms and effects of GLP-1R agonists in AD.

## Pathogenesis of AD

### Amyloid β plaque

Amyloid beta (Aβ) is a peptide with a molecular weight of 4 KDa and a length of 40-42 amino acids (3, 29). Aβ-40 and Aβ-42, obtained by hydrolysis of transmembrane amyloid precursor protein (APP) by secretases (α, β and γ), are the main types of amyloid proteins and components of Aβ plaques through the aggregation of soluble oligomers (30). Aβ-42 aggregates and combines to Ca^2+^ channels and AMPA (α-amino-3-hydroxy-5-methyl-4-isoxazolepropionic acid) receptors, to which the neurotransmitter glutamate binds, so Aβ is the most neurotoxic form (3, 31). Aβ plaques, which form and deposit in different regions of the brain, are recognized as foreign material by the brain and trigger an inflammatory and immune response by activating the microglia leading to neuronal degeneration and synaptic damage (2, 30). The amyloid hypothesis is the prevalent theory of AD pathogenesis, driven through an imbalance between Aβ production and Aβ clearance (2, 6, 21). Clinical studies have been reported that anti-Aβ protofibril antibody lecanemab has produced modest but highly statistically significant results in a trial. The drug met its primary endpoint of slowing cognitive decline in individuals with mild cognitive impairment (MCI), a clinical presentation thought to be a precursor to AD, and those with mild AD (32, 33).

### Neurofibrillary tangles

Tau is a microtubule-associated protein and mainly found in the axonal compartment (34). Tau protein interacts with tubulin to stabilize the structure of neuronal microtubules through its isoforms and phosphorylation, supporting neurite differentiation and growth, as well as transporting motor proteins along the axons (29, 34). One of the defining pathological features of AD is the intraneuronal accumulation of NFTs (35). NFTs are primarily composed of paired helical filaments consisting of hyperphosphorylated tau protein (2, 35). The affinity of hyperphosphorylation of tau protein to microtubules decreased, and hyperphosphorylated tau protein can no longer perform the function of maintaining the structure of the neurons, which slows the axonal transport (30). The current studies suggest a reduced ability to clear out misfolded, oligomerized and aggregated tau proteins that increase with advancing age (36, 37).

### Synaptic dysfunction and neurotransmitter imbalance

It has been well established that cholinergic transmission is essential for memory, learning, attention, and other higher brain functions, so cholinergic deficits play a key role in the neuropathology of AD (38). “Cholinergic hypothesis” states that the degeneration of basal forebrain neurons in AD patients leads to dysfunction and death of cholinergic neurons in the forebrain, followed by extensive presynaptic denervation occurs, and the loss of specific subtypes of acetylcholine receptors, leading to cognitive decline in AD patients (39). Acetylcholine is a major neurotransmitter in the brain, promoting experience-induced neuroplasticity, the synchronization of neuronal activity, and network connectivity (38). After depolarization of presynaptic neurons, acetylcholine is released into the synaptic cleft and binds to postsynaptic receptors such as acetylcholine muscarinic receptors (mAchRs) or acetylcholine nicotinic receptors (nAchRs) (40). Some facts attested that a reduction in the number of mAchRs and nAchRs in basal forebrain cholinergic neurons, and a 40%-50% decrease in cerebral acetylcholine level are considered pathogenic elements for dementia and AD (41, 42). Moreover, the levels of 5-hydroxytryptamine, γ-aminobutyric acid (GABA) and their receptors, as well as glutamate receptors are reduced in patients with AD (42–46). An imbalance of any of these neurotransmitters may lead to further deterioration of AD. Thus, homeostasis of multiple neurotransmitters is critical to keep cognitive integrity.

### Neuroinflammation

Neuroinflammation, microglia activation in response to amyloid deposition, plays a central role in the pathogenesis of AD (30). During acute inflammation caused by Aβ build-up, microglia phagocytose Aβ and protect neurons from Aβ toxicity (47). Under normal circumstances, acute inflammation is followed by regression through the anti-inflammatory effects of microglia. In AD, Aβ accumulation still exists, resulting in chronic neuroinflammation (48). Chronic neuroinflammation is observed at relatively early stages of disease. Chronic activation of microglia is associated with protein degradation, mitochondria dysfunction, and defects of axonal transport and apoptosis, which adversely affect neuronal function and lead to cell death (49). In addition, neuroinflammation causes immune cells (monocytes and lymphocytes in the blood, such as T cells and B cells) to infiltrate the central nervous system from the periphery across the BBB, accelerating neuroinflammation and neurodegeneration (49, 50).

### Insulin resistance

For the past few years, AD has also been considered as “type 3 diabetes” because of insulin resistance (IR) and dysregulation of insulin signaling in the brain (51). The majority of insulin in brain derives from pancreatic β-cells, which ***is*** mainly transported across the BBB. While some insulin molecules may be locally synthesized and released by neurons in the CNS (such as the hippocampus, prefrontal cortex, but not glial cells) (52). In the CNS, insulin contributes to synaptic maintenance, neuronal growth and survival, maintenance and regulation of learning and memory (53). Significantly decreased insulin and insulin receptor expression was observed in postmortem AD brain tissue, and changes in downstream insulin signaling molecules, including decreased levels of IRS-1/2, PI3K, p-Akt (51, 54). It has been reported that spatial memory improves after insulin injection in the hippocampus or intranasal administration of insulin (55, 56). Indeed, acute elevated peripheral insulin levels may increase insulin in cerebrospinal fluid, whereas, chronic peripheral hyperinsulinemia (such as insulin resistance or T2DM) may downregulate insulin receptors of the BBB, impair brain insulin uptake, and ultimately lead to learning, memory, and cognitive deficits (14, 52).

### Metabolic impairment

The brain is an organ that requires a lot of glucose to produce energy, with almost 70 percent of the energy used by neurons. In patients with T2DM, cognitive deficits are classified into three broad stages according to severity: diabetes-related cognitive decline, mild cognitive impairment (MCI) and dementia (57). MCI is a high-risk condition for conversion to AD. A large proportion of patients progress to AD, while some MCI patients may remain stable (58). All neurodegenerative diseases exhibit significant metabolic impairment, including decreased glucose uptake or utilization, with a consequent diminution in ATP production (59). Reduced glucose metabolism in AD may be a consequence of reduced postsynaptic neurotransmission, since depolarizing agents trigger glucose uptake in the brain and the effect is reduced in AD (1). In the early pathological state of AD, glucose utilization (up to 45%) is reduced, which is closely related to the alteration of insulin signaling. It has been reported that poor glucose utilization and insulin resistance can enhance Aβ deposition and decrease its clearance (60). Besides, brains with AD have been found to have a higher incidence of abnormal lipid metabolism, which is the early risk factor for the development of amyloid pathology (61). Lipids (cholesterol and sphingolipids, especially) are the main structural components of the brain, and each type of lipid may have specific function (62). Evidence shows that the strongest genetic risk factor for late-onset AD is the E4 allele of the cholesterol transporter APOE (APOE4), besides, APOE4 could promote amyloid aggregation and impair clearance from the brain directly binding to Aβ (63). Furthermore, impaired brain cholesterol synthesis can also enhance insulin resistance in brain tissue by disrupting the conformation of insulin receptor in cell membranes and promoting aberrant receptor activation (64).

### Oxidative stress and mitochondrial dysfunction

Oxidative stress is a serious imbalance between the production of reactive oxygen species (ROS) and reactive nitrogen species (RNS) and antioxidant defenses (65). Due to the active metabolism of neurons, the demand for oxygen is high and a large number of ROS are produced. Therefore, the neurons in brain are susceptible to oxidative damage and mitochondrial dysfunction (29, 66). In mouse models and autopsy analysis of AD patients, mitochondrial dysfunction and increased reactive oxygen species enhance Aβ aggregation. Moreover, the elevated markers of oxidative stress precede Aβ deposition and NFTs, suggesting that oxidative stress is an early event in the pathogenesis of AD (67). Oxidative stress raises intracellular free Ca^2+^ levels, which may have deleterious consequences. In addition, oxidative DNA damage can interfere with gene transcription and effect promoter function, resulting in transcription damage and mutation of key genes. Oxidative RNA damage can impair protein translation, and damaged RNA can degrade prematurely, further impairs the synthesis of essential proteins (65).

Regarding mitochondrial dysfunction involved in AD pathophysiology, it includes disturbances in oxidative phosphorylation (OXPHOS), and impaired energy metabolism as well as excess generation of ROS, altered mitochondrial biogenesis, transport and dynamics (68). The susceptibility of neurons to mitochondrial dysfunction may be explained by the high dependence of neurons on oxidative phosphorylation (69). In the early stages of AD, mitochondria are unable to produce enough energy due to Aβ peptides and phosphorylation of tau, and therefore impaired mitochondria eventually cause excessive production of ROS (70). Finally, it further promotes the progress of AD (71).

### Autophagy

Autophagy is an important pathway in removing abnormal protein aggregates in cells and plays an essential role in protein homeostasis (72). The mammalian nervous system, especially for neurons, depends heavily on autophagy to clear large amounts of insoluble protein aggregates to maintain protein homeostasis (73). Data shown that dysfunction of autophagy lysosome system can affect the clearance of Aβ peptides and tau proteins, two major features of AD (74). Recently, increasing evidence indicates that functional autophagy is required for synaptic functions, including neurotransmission and synaptic plasticity (75). Moreover, impaired autophagy is associated with sustained inflammation in tissues and may contribute to the pathogenesis of chronic inflammation (76). Therefore, impaired autophagy may contribute to the AD pathogenesis (73). On the other hand, phosphorylated tau protein and Aβ disturb autophagy, which is a major event in AD pathogenesis (77).

## Diagnostics and treatments of AD

Due to severe cognitive impairment in patients with advanced AD, the cost of treatment and care seriously affects economic development (2, 3). Therefore, early diagnosis and treatment are essential to stop the progression of the disease. Currently, the diagnosis of AD mainly depends on cognitive tests, imaging techniques and analysis of CSF protein. Imaging techniques are used as positive support to confirm the clinical diagnosis of AD, including MRI scans and positron emission tomography (PET) (78). Examination of CSF for p-tau, Aβ42 and total tau protein content has value in predicting AD (2, 79). However, the existing biomarker tests are either expensive or invasive, so, to develop the ideal biomarker tests for AD diagnosis is still needed (80).

**Figure 1:**
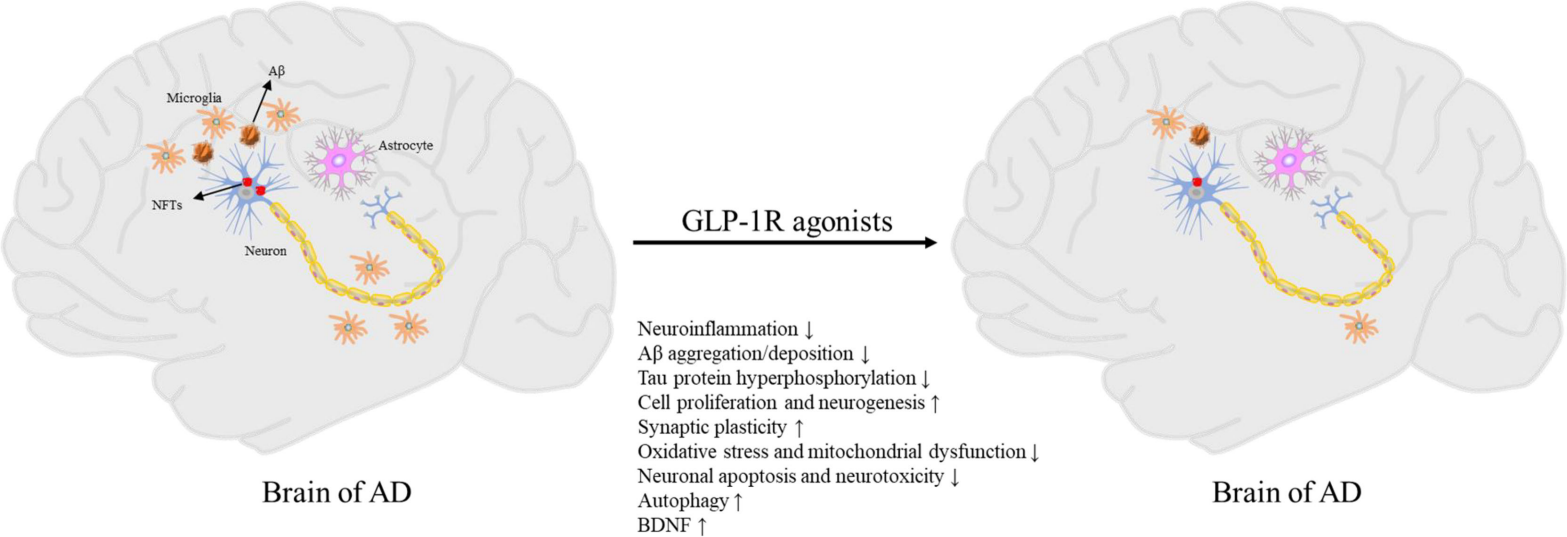
Pathogenesis of AD and neuroprotective effects of GLP-1R agonists.

Currently, there is only two classes of drugs approved for the treatment of AD: N-methyl _D_-aspartate (NMDA) antagonist (memantine), and cholinesterase inhibitors (tacrine, Donepezil, galantamine, rivastigmine). However, all these treatments are symptomatic and cannot prevent or reverse AD pathology (81). Therefore, the development of effective disease modification therapies is the focus of AD prevention and treatment research in the future. Due to the similar molecular mechanism between T2DM and AD, several drugs for the treatment of T2DM are increasingly being proposed for the treatment of AD (15, 82). Some drugs for the treatment of T2DM may cause systemic side-effects such as hypoglycemia, and the long-term administration of insulin could promote brain insulin resistance (17). In contrast, GLP-1R agonists are safer, and several studies have shown that GLP-1R agonists significantly improve cognitive impairment (**Figure 1**) (25, 26, 83–85).

## Biological characteristics of GLP-1

In the 1980s, a new glucagon-like peptide, produced from proglucagon cleavage, was discovered to stimulate insulin secretion (86). Two glucagon-related peptides were identified in the proglucagon sequence, and were named glucagon-like peptides 1 and 2 (GLP-1 and GLP-2) (87). However, neither GLP-1 nor GLP-2 were active on insulin secretion, but a truncated version of GLP-1 was subsequently found to enhance insulin secretion in various experimental models and human studies (88–90). Ultimately, GLP-1 was identified as a potential incretin hormone (89, 91).

GLP-1, a 36-amino acid peptide, is produced in enteroendocrine L-cells of the distal small bowel and colon (92). Besides, GLP-1 can also be produced by neurons in CNS (20, 93). GLP-1 has different forms include GLP-1(1-37), GLP-1(7-36)amide and GLP-1(7-37) (22, 89). In humans, nearly all circulating GLP-1 is one of the latter two forms (89). While GLP-1(7-36)amide and GLP-1(7-37) are equally effective in stimulating insulin and C-peptide secretion, GLP-1(1-37) is much lower insulinotropic efficacy (88, 90, 94).

The effect of GLP-1 depends on blood glucose levels since it can only potentiate glucose-stimulated insulin secretion from islet beta cells in the hyperglycemic state rather than in the normal blood glucose state (95, 96). In addition, GLP-1 inhibits glucagon secretion in islet alpha cells, but only when blood glucose levels are higher than fasting (95, 96). Therefore, GLP-1 is save for use in T2DM and non-diabetic patients (97). Due to DPP-4 and renal clearance, native GLP-1 in humans has a half-life of about 1-2 minutes (21, 22, 89). In addition, DPP-4 cleaved GLP-1(7-36)amide and GLP-1(7-37) to form GLP-1(9-36)amide or GLP-1(9-37), low-affinity ligand of GLP-1 receptor and nonprimary role in regulating glucose metabolism (98–101). DPP-4 inhibitors, such as sitagliptin, alogliptin, linagliptin and saxagliptin, were synthesized to prolong the half-life of GLP-1 (102). Although DPP-4 inhibitors do not cross the BBB under normal conditions (the pharmacokinetic feature may prevent their repurposing use in neurodegenerative diseases), the permeability of BBB is increased in neurodegenerative diseases, so molecules otherwise unable to cross BBB could enter into CNS in these conditions (103). Several studies have showed the neuroprotective effects of DPP-4 inhibitors in animal models of AD (104, 105). Therefore, DPP-4 inhibitors, like GLP-1R agonists, may be a potential drug for the treatment of AD.

## Tissue distribution of GLP-1R

GLP-1 mediates its effects by binding to its receptor, the GLP-1R, which is a sevenfold transmembrane G-coupled receptor that increases levels of cAMP by activating adenylate cyclase (89, 95, 96). GLP-1R is abundantly present in the pancreatic beta cells, gut, and the CNS, including the cerebral cortex, hippocampus, hypothalamus, thalamus, caudate-putamen and globus pallidum (89, 106–108). In addition, GLP-1R is moderately in the lung, heart, kidney, blood vessels, pancreatic alpha cells, and peripheral nervous system, with no expression of GLP-1R in liver, skeletal muscle or adipose tissue (109–112). However, the analysis of GLP-1R always confronted an obstacle due to the absence of antibodies with sufficient selectivity and availability. Further, it is worth noting that there was disputed information in the expression of GLP-1R in different cell types. The exact cellular localization of the GLP-1R remains equivocal due to the lack of selective antibodies and application of specific anti-GPCR antibodies (22). Recent studies identified the expression of GLP-1R in adipocytes (22, 113). In addition, low expression of GLP-1R in liver and muscle has been proposed (113).

In the peripheral system, GLP-1R mediates the actions of GLP-1 *via* the incretin axis, in which stimulation of the GLP-1R with GLP-1 primarily triggers insulin release from islet β cells in a glucose-dependent manner and inhibits glucagon secretion from islet α cells (110). The GLP-1R transduces signal mainly through G_αs_ coupling pathway (114). GLP-1R signaling enhances glucose-dependent insulin secretion by activating of G_αs_, upregulating of cAMP, and subsequently activating of PKA (110). The cAMP/PKA pathway inhibits voltage-gated potassium channels that respond to depolarization, opens and allows K^+^ efflux, repolarizing the cell and allowing increased calcium influx through voltage-dependent calcium channels, resulting in the exocytosis of insulin from β-cells (115). GLP-1R activity also promotes the transactivation of epidermal growth factor receptor (EGFR), which then signals through phosphoinositide 3-kinase (PI3K) and insulin receptor substrate-2 (IRS-2), and subsequently activates extracellular-signal-regulated kinase 1 and 2 (ERK1/2) and nuclear translocation of PKC ξ to mediate β-cell proliferation and differentiation (110, 114).

In the CNS, GLP-1 also exert neuroprotective and neurotropic effects by binding to GLP-1R (20, 22, 116). GLP-1R expressed in the nucleus tractus solitarius signals to suppress appetite, delay gastric emptying and reduce body weight, and this is proposed to be mediated through PKA, decreasing phosphorylation of AMPK (22, 89, 110). In addition, GLP-1R overexpression results in improved cognitive function in mice and GLP-1R knockout severely impairs cognitive ability (5, 117, 118).

## GLP-1R agonists

**Figure 2:**
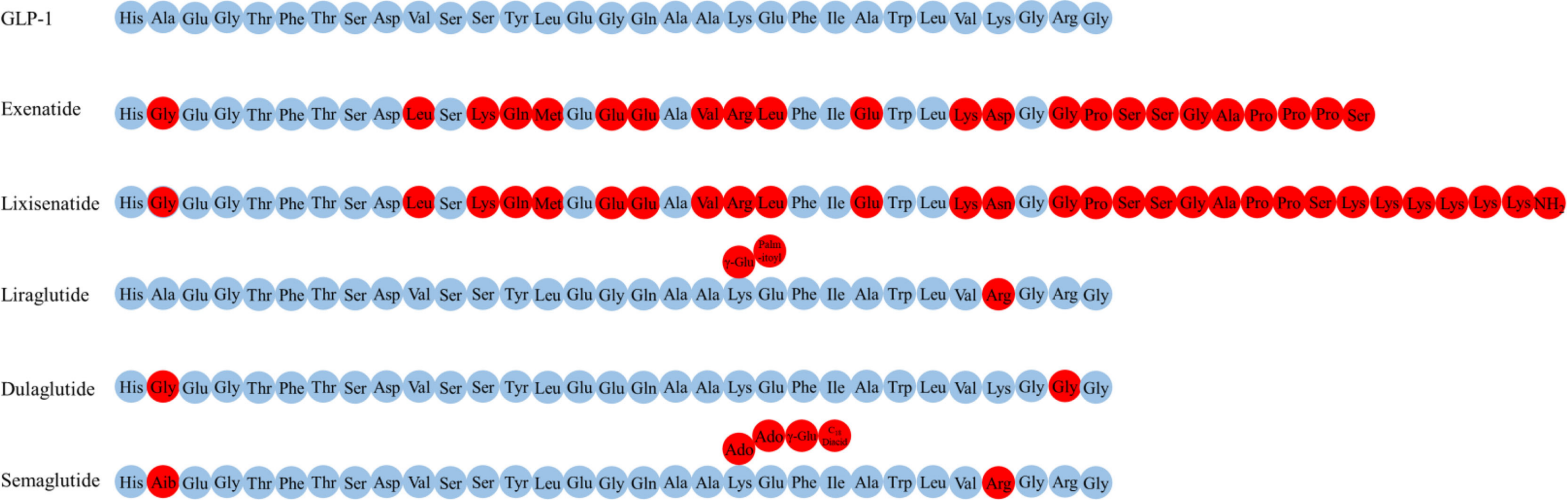
The amino acid structure of various GLP-1R agonists.

Several GLP-1R agonists, which mainly delay protease DPP-4 metabolism to overcome the problem with the rapid inactivation of GLP-1, have been developed (5, 119). Currently approved and commonly used GLP-1R agonists include exenatide (the synthetic form of Ex-4, a naturally occurring GLP-1R agonist), lixisenatide, dulaglutide liraglutide and semaglutide (**Figure 2**) (17, 110).

Exenatide, a peptide of 39 amino acids, has a much slower metabolism than endogenous GLP-1 (half-life of 3-4 h) (119, 120). Exenatide readily enters the brain when injected intravenously into mice (102). Elimination of exenatide is primarily achieved by glomerular filtration with subsequent proteolytic degradation (120–122). Increasing the dose of exenatide is not recommended in patients with an estimated glomerular filtration rate (eGFR) of 30-60 mL/min/1.73 m^2^, it is contraindicated for use in patients with an eGFR of less than 30 mL/min/1.73 m^2^ (123).

Lixisenatide comprises 44 amino acids and is based on the Ex-4 peptide sequence, omitting proline at position 36 and adding six lysine residues at the C-terminal (110). The binding affinity of lixisenatide to GLP-1R is fourfold higher than native GLP-1 (17). The circulating half-life of lixisenatide is approximately 3 h (124). Studies have shown that significant concentrations of lixisenatide were found in the brain of mice 30 minutes and 3 hours after intraperitoneal injection, suggesting that lixisenatide could cross the BBB (125). Lixisenatide is cleared by glomerular filtration, followed by tubular reabsorption and subsequent metabolic degradation (126). Dose adjustment is not recommended in patients with mild-severe renal impairment (eGFR = 15-89 mL/min/1.73 m^2^), but use is not recommended for patients with end-stage renal disease (127).

Liraglutide, 97% sequence identity to native GLP-1, is obtained by derivatizing GLP-1 with a fatty acid (128). This also facilitates albumin-binding and DPP-4 resistance, thereby allowing a half-life of 13 h (129). The main mechanisms of liraglutide protraction are as follows: (1) slow absorption after subcutaneous injection (130); (2) reduce clearance rate due to slowed metabolism and renal filtration (131). Significant levels of liraglutide were found in the brain of mice 30 minutes and 3 hours after intraperitoneal injection, indicating that liraglutide could cross the BBB (125). Liraglutide is approved in Europe for patients with T2DM and mild or moderate kidney damage, but is currently not recommended or should be used with caution in patients with severe renal impairment (130).

Dulaglutide is a long-acting GLP-1R agonist with a half-life of 4 days (124). The dulaglutide molecule consists of two modified DPP-4 resistant GLP-1(7-37) peptides fused to a modified IgG4 Fc fragment, which protect dulaglutide from proteolytic degradation by DPP-4 (132). In addition, its high molecular weight (57 kDa) prevents renal clearance and prolongs its half-life (133). No studies have addressed whether dulaglutide could cross the BBB. In patients with varying degrees of renal or hepatic impairment, no relevant change in dulaglutide exposure was observed relative to the degree of renal or hepatic impairment (132). Moreover, use of dulaglutide in people with T2DM is likely to confer additional renal benefits, and dulaglutide may be used in advanced chronic kidney disease at any eGFR level and without dose adjustment in contrast with most other noninsulin diabetes therapies (134, 135).

Semaglutide, a type of GLP-1R agonists with 94% sequence homology to GLP-1 and with an extended half-life of approximately 1 week, has been clinically approved to treat T2DM and is available in subcutaneous and oral dosage form (136, 137). Semaglutide interacted with circumventricular organs, where GLP-1R expression is abundant, and with regions protected by the BBB (lateral septal nucleus, nucleus tractus solitarius, hypothalamic arcuate nucleus) (102). The main elimination routs of semaglutide are through urine and feces. About 3% of the dose is emitted in the urine in an integral form (136).

## Neuroprotective effects of GLP-1R agonists

**Figure 3:**
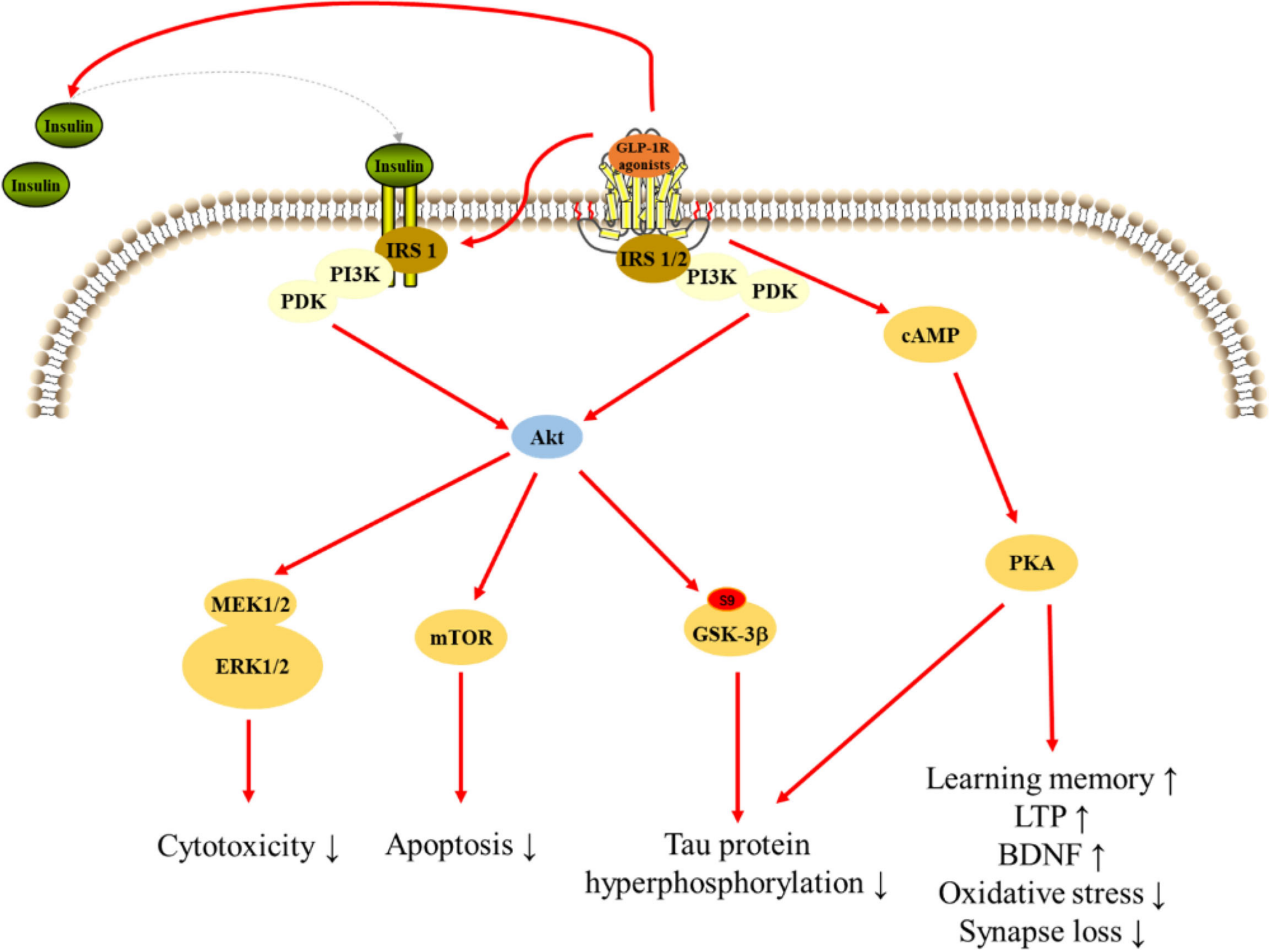
Signaling pathways for GLP-1R agonists in regulating cellular events in AD.

GLP-1 and its mimetics can cross the BBB, therefore, are able to affect the CNS function such as cognition and neuroprotection (138). GLP-1R agonists were initially used to treat T2DM and soon after it was found that these drugs possess many other physiological properties, such as neuroprotection, neurotrophic, and anti-inflammatory effects, which may be useful to slow the progression of AD (**Figure 3**) (17).

### Anti-inflammatory effects

Previous studies have shown that GLP-1R mimics have anti-inflammatory effects in the CNS (139–141). Parthsarathy et al. found that liraglutide, an GLP-1R agonists, reduces the activated microglia load in the cortex and dentate gyrus region of hippocampus, and the activated astrocyte load in the cortex. Furthermore, the pro-inflammatory cytokine levels of IL-6, IL-12p70, IL-1β, and total nitrite concentration are reduced in the brains of mice treated with liraglutide (142). A study reports that prophylactic liraglutide treatment reduces chronic inflammation (activated microglia) in the cortex and prevents memory impairment in APP/PS1 mice (143). In a number of studies, GLP-1R agonists such as liraglutide, exenatide and lixisenatide can reduce neuroinflammation in AD models, thereby improving cognitive dysfunction (140, 141, 144–148). These data strongly suggest that GLP-1R agonists are beneficial to attenuate neuroinflammation-associated cognitive impairment and thereby improve cognition.

### Reducing Aβ aggregation/deposition and tau protein hyperphosphorylation

Some studies have reported that GLP-1 can affect the pathological process of Aβ deposition and tau hyperphosphorylation (149–151). Perry et al. found that GLP-1 can reduce the levels of endogenous Aβ in the brain and reduce the levels of APP in cultured neuronal cells (152). Exendin-4 (an endogenous insulin releasing incretin, GLP-1) reduces Aβ accumulation and tau hyperphosphorylation in cellular and animal models of AD (85, 151, 153–156). In another study, (Val^8^)GLP-1 might prevent age-related neurodegenerative changes (such as AD) by preventing decline of learning and memory formation, reduction of tau hyperphosphorylation and protection of subcellular structures and morphology of neurons (149). Lixisenatide, a GLP-1R agonist, reduces neurofibrillary tangles and amyloid plaque load, and thereby ameliorates learning memory deficits in APP/PS1 mice (144, 157). Total brain APP and Aβ oligomer levels are reduced in Liraglutide-treated AD mice (147, 158–160), and intervention with liraglutide can prevent tau hyperphosphorylation (161–164). Dulaglutide, a novel long-acting GLP-1R agonist, ameliorates AD-like impairment of learning and memory ability by decreasing the hyperphosphorylation of tau and NFs proteins (150). Therefore, GLP-1R agonists might improve cognitive and memory function by reducing Aβ deposition and tau hyperphosphorylation in the brain.

### Promoting cell proliferation and neurogenesis

In addition to its hormonal and neuropeptide activity, GLP-1 also is considered a growth factor that regulates cell growth and differentiation, and promotes interruption of pro-apoptotic processes (165). Hamilton et al. have found that progenitor cell division is enhanced after injected subcutaneously GLP-1 agonists exenatide (exendin-4) and liraglutide in the dentate gyrus of brain of mouse models of AD (166). In another study, intraperitoneal injection with GLP-1 analogue (Val)GLP-1 enhances neuronal stem cells and neurogenesis in the dentate gyrus of brain in wild type mice, so it may have potentially beneficial effects in the CNS (167). Moreover, chronic treatment with liraglutide showed an increase in stem cell proliferation and differentiation into mature neurons in APP/PS1 mice and controls at all ages, which may have beneficial effects in neurodegenerative disorders like AD (168). And total hippocampal CA1 pyramidal neuron numbers in senescence-accelerated mouse prone 8 mice are increased after received liraglutide, at the same time, the memory retention of mice is increased (169). Overall, GLP-1 may improve cognitive function by promoting neuronal stem cell proliferation and differentiation.

### Enhancing synaptic plasticity

Evidence shows that the changes in synaptic function may be an early event in AD pathogenesis (170). Studies have found that (Val^8^)GLP-1 protects synapses from the detrimental effects of Aβ fragments on synaptic plasticity formation (171–173). In another study, McClean et al. reported that GLP-1R analogues enhance synaptic plasticity in area CA1 of the hippocampus (174). However, synaptic plasticity and memory formation are impaired in GLP-1 receptor knockout mice (118). Liraglutide was shown to prevent synapse loss and deterioration of synapse plasticity in the hippocampus of APP/PS1 mice, while enhance synaptic plasticity in the control mice (158, 175). And liraglutide protects synapse from Aβ oligomers-induced damage in hippocampal neurons (176, 177). Ohtake et al. found that exendin-4 increased the membrane protein level of the AMPA receptor GluR1 subunit and postsynaptic density protein-95, which were the critical mechanisms of long-term potentiation (LTP) as well as the formation of learning and memory (178). Moreover, lixisenatide and GLP-1 analogue CJC-1131 could protect against Aβ-induced suppression of hippocampal LTP (179, 180). These results suggest that one of neuroprotective effects of GLP-1R agonists is enhancing synaptic plasticity.

### Attenuating oxidative stress and mitochondrial dysfunction

Oxidative stress plays a vital role in the pathogenesis and pathophysiology of AD (181). Chen et al. have reported that GLP-1/exendin-4 could ameliorate oxidative stress-induced injury in PC12 cells (182). GLP-1 also protects HT22 cells against oxidative stress-induced cell death (183). Moreover, Spielman et al. found that GLP-1 can reduce oxidative stress in BV2 microglia by inhibiting the accumulation of intracellular ROS and release of nitric oxide (NO), as well as by increasing the expression of the antioxidant glutathione peroxidase 1 (GPx1) and superoxide dismutase 1 (SOD1) (184). Studies have demonstrated that Liraglutide has neuroprotective effects on AD-like neurodegeneration induced by H_2_O_2_ in human neuroblastoma cell line SH-SY5Y (185, 186), and protects against brain Aβ accumulation by partially rescuing oxidative stress (146).

Mitochondria are involved in a series of biochemical events in cells, ordinarily, normal neurons have an intense energetic demand to support localized neuronal activities. However, dysfunctional mitochondria are associated with impaired neuronal function and associated neurodegenerative diseases (187). According to the report of An et al. GLP-1R agonists could repair mitochondrial damage *in vitro*, and promote mitochondrial biogenesis and antioxidant system peroxisome proliferator-activated receptor γ coactivator 1α (PGC-1α) signaling pathway *in vivo* (28). Xie et al. have reported that Liraglutide ameliorates mitochondrial dysfunction and prevents neuronal loss in the brain of 5×FAD mice (188). Likewise, another study shows that Exenatide alleviates mitochondrial dysfunction and cognitive impairment in 5×FAD mice (84). Moreover, Exendin-4 significantly increases Aβ-induced decrease in mitochondrial function, integrity and respiratory control rate in all brain regions (189).

These results suggest that GLP-1R agonists are able to improve cognitive function by attenuating the oxidative stress and mitochondria dysfunction in CNS.

### Inhibiting neuronal apoptosis and neurotoxicity

Neuronal apoptosis, induced by Aβ and stress, is regard as a physiolopathologic marker in AD brain (190). Perry et al. found that GLP-1 and exendin-4 could completely protect cultured rat hippocampal neurons against glutamate-induced apoptosis (191). Moreover, exendin-4 protected PC12 cells from Aβ-induced apoptosis (192). During et al. reported that [Ser (2) exendin (1-9)], a GLP-1R agonist, significantly attenuated kainic acid-induced apoptosis in the CA3 region of the hippocampus (117). In another study, GLP-1 protected against methylglyoxal-induced pheochromocytoma (PC12) cell apoptosis though the PI3K/Akt/mTOR/GCLc/redox signaling pathway (193). Consistent with these data, GLP-1 attenuated apoptosis of PC12 cells induced by carboxymethyl lysine *via* peroxisome proliferation activated receptor-γ (PPAR-γ) (194). Chen et al. demonstrated that GLP-1 could significantly decrease the percentage of advanced glycation and products (AGEs)-induced SH-SY5Y cell apoptosis (195). And it also has been reported that liraglutide reduces cytotoxicity and apoptosis of SH-SY5Y cells during methylglyoxal, thapsigargin and Aβ stress (196–199). Likewise, in an AD model, SH-SY5Y cells were treated with Aβ, semaglutide inhibited apoptosis by inhibiting the expression of Bax induced by Aβ and increasing the expression of Bcl2 inhibited by Aβ (200). In summary, these data suggest one of neuroprotective effects of GLP-1R agonists is inhibiting neuronal apoptosis.

### Enhancing autophagy

Autophagy deregulation may underlie the accumulation of neuropathological markers for AD, rendering it one of the main features in AD (201). Candeias et al. found that peripheral exendin-4 treatment promoted brain cortical autophagy upon type 2 diabetes rats (202). Liraglutide modulated the autophagy machinery homeostasis in SH-SY5Y cells (198), and attenuated Aβ42 generation in SH-SY5Y cells through enhancing autophagy (160). Another study showed that semaglutide protected against Aβ_25-35_ in SH-SY5Y cells by enhancing autophagy (200). These indicate autophagy is a key molecular event for GLP-1R agonists to protect neurons against damage.

### Other effects

In addition to above effects, GLP-1R agonists have other neuroprotection mechanism on CNS, such as increasing the brain-derived neurotrophic factor (BDNF) levels and activity, regulating calcium homeostasis, promoting glycolysis, and reducing vascular damage. BDNF plays a crucial role in the pathophysiology of brain neurons. Ohtake et al. found exendin-4 increased the expression of BDNF in mouse neocortex (178). And several studies suggest that exenatide increased expression levels of BDNF in AD mice hippocampus and cortex (141, 203, 204). It has been shown that intracellular calcium overload induced by Aβ produces cytotoxicity, which cause a decrease in learning and memory as well as cognitive function. Recent studies reported that GLP-1R agonists, such as Val^8^-GLP-1(7-36), exendin-4 and liraglutide, attenuated Aβ_1-42_-induced calcium overload by regulating intracellular calcium homeostasis in cortical or hippocampal pyramidal cells (173, 205, 206). Glycolysis and oxidative phosphorylation, which break down glucose into the form of ATP to produce energy, is the main source of energy for the brain. Bomba et al. found that exenatide increased brain lactate dehydrogenase activity, enhancing anaerobic glucose catabolism in brains of PS1-KI mice (207). Zheng et al. revealed liraglutide improved aerobic glycolysis in astrocyte, and cortices of 5×FAD mice (208). Cerebral microvascular impairments occurring in AD may reduce Aβ clearance. Some studies found liraglutide reduced incidence of cerebral microanuerysms and leakage (209, 210).

Above all, GLP-1R agonists may improve cognitive function *via* different mechanism. These results suggest that GLP-1R agonists may have therapeutic and preventive effects on AD.

## Signaling pathways underlying the neuroprotective effects of GLP-1R

### PI3K signaling pathway

PI3K plays an important role in modulating cytoactivities. Data showed that GLP-1 significantly increased PI3K, Akt, and mammalian target of rapamycin (mTOR) phosphorylation without inducing the expression of PI3K, Akt, or mTOR. And the expression of downstream gene significantly reduced after treated inhibitors of PI3K, Akt, and mTOR. These results suggest PI3K/Akt/mTOR signaling mediated the protection of GLP-1 on apoptosis in neurons (193). Exendin-4 treatment reversed the intracerebroventricular-streptozotocin (ICV-STZ) -induced decline in the levels of phosphorylation of Akt at Ser473 and glycogen synthase kinase 3β (GSK-3β) at Ser9, a key kinase in AD, leading to decrease hyperphosphorylation of tau in rat hippocampus (182). And, exendin-4 significantly increased Aβ-induced decrease in the level of phosphorylated Akt in brain (189). However, lixisenatide inhibited the Aβ-induced activation of GSK-3β, with a significant increase in the phosphorylation of ser9 and a significant decrease in the phosphorylation of Y216, suggesting that lixisenatide can prevent Aβ-related impairments by affecting the PI3K-Akt-GSK3β pathway (179). Liraglutide administration prevented the decrease of AKT and GSK-3β phosphorylation after treated Aβ_1-42_ protein, which inhibiting tau hyperphosphorylation (164, 211). Moreover, liraglutide prevents Aβ and H_2_O_2_-induced neurotoxicity in SH-SY5Y cells *via* PI3K/Akt signaling pathway (185, 197). Lixisenatide relieved the Aβ_25-35_-induced suppression of the phosphorylation of Akt and MEK1/2 indicating the neuroprotection of lixisenatide might related to the Akt-MEK1/2 signaling pathway (206). A study reported that GLP-1(7-36) could protect HT22 cells against stressors *via* activation of survival signaling molecules, such as Akt and ERK1/2 (183). Dulaglutide decreased the hyperphosphorylation of tau and NFs proteins through improving the PI3K/Akt/GSK3β signaling pathway, which may be related to its protective effects on impaired of AD-like learning and memory (150).

### cAMP/PKA pathway

The cAMP/PKA pathway, a ubiquitous cascade that modulates numerous cellular events within neurons (176). Pretreatment with liraglutide effectively and dose-dependently protected against the Aβ_25-35_-induced impairment of spatial memory and deficit of late-phase long-term potentiation (L-LTP), and also activated cAMP signal pathway in the rat brain (177). Furthermore, pretreatment of cultures with liraglutide attenuated measures of synapse induced by Aβ oligomers (AβOs), but liraglutide failed to prevent AβOs-induced synapse loss after treated PKA inhibitor, and liraglutide attenuated the AβOs-induced decrease of PKA activity, suggesting that activation of the cAMP/PKA pathway underlies the neuroprotective actions of liraglutide (176). Besides, GLP-1R agonists also regulated PC12 cells growth through regulating cAMP/PKA signaling pathway (212). In addition to acting on neuronal cells, GLP-1R agonists also exert effects on microglia and astrocytes through cAMP/PKA signaling pathway. GLP-1 reduced apoptotic death of BV2 microglia cells through the binding and activation of the GLP-1R, with subsequent activation of the PKA pathway. Moreover, GLP-1 upregulated BV2 microglia cells expression of BDNF in a PKA-dependent manner (184). In Aβ-treated astrocytes, GLP-1 prevented mitochondrial fragmentation, and improved the neuronal supportive ability *via* cAMP/PKA pathway (188).

### Insulin signaling pathway

Insulin signaling in the brain is vital for the brain activity, and insulin resistance is one of key reasons of AD (213). Liraglutide treatment significantly decreased insulin receptor aberrations in conjunction with a concomitant decrease in amyloid plaque load and a highly significant reduction in astrocytosis and microglial number associated with both plaques and IR pathology (214). Liraglutide also restored neuronal insulin sensitivity in hyperinsulinemic conditions and reduced the Aβ formation and tau hyperphosphorylation in neuronal cells (215). The ERK and JNK are parts of the insulin signal pathway and closely associated with tau hyperphosphorylation. Liraglutide ameliorated the phosphorylated expressions of ERK and JNK interfered with by STZ and decreased the hyperphosphorylation of NFs (216). Exendin-4 significantly increased insulin level and phosphorylation of insulin receptor substrate 1 (IRS-1) in rat hippocampus, and could not change the status of tau phosphorylation without insulin in HT22 neurons, suggesting that insulin is required in reduction of tau hyperphosphorylation by GLP-1R agonists (154).

## Application of GLP-1R agonists in AD

Several studies have reported that GLP-1 agonists could protect brain against various damage in AD models (**Supplementary Table 1** and **Table 1**). Currently, GLP-1R agonists used in the treatment of AD models include (Val8)GLP-1, GLP-1(9-36)amide, GLP-1(7-36)amide, CJC-1131, Geniposide, Exendin(5-39), Exendin-4, NLY01 (engineered exendin-4), liraglutide, Lixisenatide, Dulaglutide, Exenatide, GLP-1, dual GLP-1/GIP receptor agonist (DA-JC4, DA-JC1, DA5-CH, DA-CH3), GLP-1/GIP/Gcg receptor triagonist (triagonist, TA), dual GLP-1 and Gcg receptor agonist (oxyntomodulin) (144, 147, 148, 150, 171, 180, 186, 189, 194, 218, 219, 228, 235, 237, 238, 241, 242).

**Table 1:** The effects of various GLP-1R agonists upon brain mechanisms in animal models for AD.

Drug	Effect of improving cognitive function	Effect of attenuating neuroinflammation	Effect of reducing Aβ aggregation/deposition	Effect of reducing tau protein hyperphosphorylation	Effect of enhancing LTP
***(Val^8^)GLP-1***	***+++***	***-***	***-***	***++***	***+++***
***GLP-1(9-36)^amide^***	***+++***	***+++***	***-***	***/***	***+++***
***GLP-1(7-36)amide***	***+++***	***/***	***/***	***/***	***+++***
***CJC-1131***	***+++***	***/***	***/***	***/***	***+++***
***Geniposide***	***+++***	***+++***	***+***	***+++***	***+++***
***Exendin-4***	***+++***	***++***	***+++***	***+++***	***+++***
***Liraglutide***	***+++***	***+++***	***+++***	***+++***	***+++***
***Lixisenatide***	***+++***	***++***	***+***	***++***	***+++***
***Dulaglutide***	***++***	***/***	***/***	***+++***	***/***
***Exenatide***	***+++***	***+++***	***+***	***-***	***/***
***dual GLP-1/GIP receptor agonist***	***+++***	***+++***	***+++***	***+++***	***+++***
***Triagonist***	***+++***	***+++***	***+++***	***+++***	***+++***
***Oxyntomodulin***	***+++***	***/***	***+++***	***/***	***+++***

/The effect of GLP-1R agonists is not mentioned;

-No effect on brain mechanism;

+The effect of GLP-1R agonists on brain mechanism is less than 50% (relative to the control group and the AD group without GLP-1R agonists);

++The effect of GLP-1R agonists on brain mechanism is between 50%-75% (relative to the control group and the AD group without GLP-1R agonists);

+++The effect of GLP-1R agonists on brain mechanism is more than 75% (relative to the control group and the AD group without GLP-1R agonists).

The treatment of (Val^8^)GLP-1 30 minutes prior to injection of Aβ fully reversed the impairment of LTP induced by Aβ (171, 217). Val8-GLP-1 also protected against Aβ_1-40_-induced impairment of learning and memory, and reduced total tau expression and hyperphosphorylated tau levels (149, 217). Likewise, GLP-1(9-36)amide could reverse AD-related alterations in hippocampal synaptic plasticity and memory deficits, but did not alter levels of APP and Aβ in APP/PS1 mice (218). And, GLP-1(7-36)amide significantly prevented LPS-, IL-1β-, H2O2-induced impairment in synaptic functions of hippocampal CA1 region (219). CJC-1131, a new chemical-modified GLP-1 agonist, was effective in resisting DPP-4 degradation. It is found that CJC-1311 effectively prevented Aβ_1-42_-induced impairments in spatial learning and memory, and also reversed Aβ_1-42_-induced suppression of hippocampal LTP (180). Geniposide, acting as a GLP-1R agonist, partially prevented STZ-induced learning and memory impairment *via* modulation of PI3K/GSK-3β signaling pathway and reduced STZ-induced hyperphosphorylation of tau protein (220). Besides, geniposide suppressed Aβ accumulation and alleviated cognitive dysfunction in APP/PS1 mice, attenuated Aβ-induced reduction of LTP in acute hippocampal slices, and attenuated Aβ-induced synaptic dysfunction in cultured hippocampal neurons (221, 222).

There are a large number of studies that have reported the application of exendin-4, liraglutide and exenatide in the treatment of cognitive dysfunction and pathological features in AD models. Exendin-4 treatment could reduce levels of Aβ in brain of mice and reduce levels of APP in cultured neuronal cells (152). Exendin-4 also could ameliorate brain levels of AβPP and Aβ, elevated by STZ-induced diabetes, in 3×Tg-AD mice (151). Behavioral measures of cognition in APP/PS1 mice treated with Exendin-4 was significantly improved. After administration of Exendin-4, Aβ oligomers-induced impaired axonal transport was prevented, and levels of soluble Aβ and amyloid plaque load were decreased in APP/PS1 mice (224, 227). In STZ-treatment rats, Exendin-4 improved learning and memory performance, protected hippocampal neurons against degeneration, and reversed STZ-induced tau hyperphosphorylation through downregulation of GSK-3β activity (154, 155, 182). For Aβ_1-42_-induced AD model, exendin-4 mitigated abnormal behavior and prevented Aβ1-42-induced impairment of LTP (85, 189, 226). Similar to exendin-4, liraglutide also improved cognitive and spatial memory, enhanced induction and maintenance of LTP, and reduced β-amyloid plaque formation as well as inflammatory response in APP/PS1 (143, 158, 159, 175, 214). Liraglutide decreased hyperphosphorylation of NFs and hyperphosphorylated tau in brain of STZ mice, and improved learning and memory impairment induced by STZ through ameliorating ERK and INK signaling pathways (216, 229). For Aβ_25-35_-treated mice, liraglutide dose-dependently prevented against Aβ_25-35_-induced impairment of spatial learning and memory, prevented depression of hippocampal L-LTP, and upregulated intracellular cAMP level (177). In db/db mice, Aβ_1-42_-treated mice or hTauP301L mice, liraglutide alleviated tau hyperphosphorylation, and prevented dysregulation of Akt and GSK-3β in brain (161, 163, 164). Liraglutide improved learning and memory performance, attenuated hyperphosphorylation of tau as well as NFs, and prevented neurodegeneration *via* improving JNK and ERK signaling in brain of 3×Tg mice (232). Liraglutide reduced astrocytes, microglia activity and amount of Aβ levels in cortical and hippocampal CA1 and CA3 regions in 5×FAD mice (147). While liraglutide improved spatial cognition, it also reduced astrocytes and microglia activity, ameliorated amount of Aβ levels and mitochondrial dysfunction in cortical and hippocampal CA1 and CA3 regions, and prevented neuron loss by activating cAMP/PKA pathway in brain of 5×FAD mice (188, 208). Lixisenatide and exenatide have the same effect as liraglutide in AD models (84, 141, 144, 148, 157, 179, 206, 213).

Moreover, dual GLP-1 and Gcg receptor agonist, dual GLP-1/GIP receptor agonist and GLP-1/GIP/Gcg receptor triagonist have also been successfully applied in the treatment of AD models. It is reported that both DA-JC4 and DA5-CH could improve STZ-induced learning and memory impairment, reduce levels of phosphorylated tau protein and STZ-induced chronic inflammation response in brain (233, 237). Besides, dual GLP-1/GIP receptor agonists (DA-JC4, DA5-CH, DA-JC1 and DA-CH3) reduced inflammation response, amyloid plaques and tau phosphorylation in brain, reversed memory loss, and enhanced synaptic plasticity in hippocampus of APP/PS1 mice (186, 234, 236, 238). In 3×Tg-AD treated with DA-JC4, the ability of recognize new object and spatial working memory was improved, hippocampal Aβ and tau pathology were alleviated, and hippocampal synaptic plasticity also was improved as well as levels of PSD95 and SYP were increased (235). After administration of Triple GLP-1/GIP/glucagon receptor agonist, learning and memory impairment of AD models (APP/PS1 mice and 3×Tg-AD mice) was improved, amyloid plaques, tau pathology, inflammation response and oxidative stress in brains of two AD models were ameliorated (239–241).

However, there are few clinical trials of GLP-1R agonists in AD patients. In a randomized placebo-controlled, double-blinded trial, although liraglutide did not affect Aβ load and cognition measures, 26 weeks of liraglutide treatment prevented expected decline of glucose metabolism that signifies cognitive impairment, synaptic dysfunction, and disease evolution (243). And the effect of liraglutide might be associated with improvement of BBB glucose transport capacity (244). Another double-blinded, placebo-controlled study reported that liraglutide increased intrinsic connectivity in bilateral hippocampal, medial frontal and lateral occipital regions, suggesting that liraglutide might reduce or delay AD progression (245). Patients with T2DM, persons at risk for AD, had a significantly lower associated risk of AD after administered exenatide, liraglutide and dulaglutide (83). These data indicate that GLP-1R agonists show great potential as a novel treatment for preventing AD processes.

## Conclusion

Increasing evidence suggests that anti-diabetes medicine, GLP-1R agonists, have multiple neuroprotective mechanisms in AD models, such as anti-inflammation, anti-oxidative stress, reducing Aβ aggregation/deposition and tau protein hyperphosphorylation, reducing neuronal apoptosis and neurotoxicity, increasing cell proliferation and neurogenesis, increasing synaptic plasticity, and other beneficial effects. GLP-1R agonists might have the potential to be developed as a novel treatment of AD. Further clinical evidence is needed to verify the effects of GLP-1R agonists on cognitive function and pathology in AD patients.

## Author contributions

JX and HD designed the study. HD wrote the paper. XM reviewed the paper and provided suggestions. YY collected references. JX is the guarantor of this work and had full access to all the data in the study and also takes responsibility for the integrity of the data. All authors contributed to the article and approved the submitted version.

## Funding

This study was supported by grants from the Scientific Research Foundation of the Ministry of Education of China (Grant No.2021JH019) and Scientific Research Foundation of Postdoctoral Department of Heilongjiang Province (Grant No.LBH-Q21025).

## Conflict of interest

The authors declare that the research was conducted in the absence of any commercial or financial relationships that could be construed as a potential conflict of interest.

## Publisher’s note

All claims expressed in this article are solely those of the authors and do not necessarily represent those of their affiliated organizations, or those of the publisher, the editors and the reviewers. Any product that may be evaluated in this article, or claim that may be made by its manufacturer, is not guaranteed or endorsed by the publisher.
