# Supplementary material for: The mechanism and efficacy of GLP-1 receptor agonists in the treatment of Alzheimer’s disease
Source: Front Endocrinol (Lausanne). 2022 Nov 17;13:1033479. doi: 10.3389/fendo.2022.1033479 (PMC9714676; doi:10.3389/fendo.2022.1033479)
Supplement: Supplementary file 1 [file Table_1.docx]

Supplementary Table 1. Studies about GLP-1R agonists’ protection of brain from various damages in animal models for AD.

| Drug | Reference | Mouse/rat line & age or weight | Definition of the model | Application of GLP-1 agonist (dose and method) | Experimental groups | Behavioral and molecular assessments | Main findings |
| --- | --- | --- | --- | --- | --- | --- | --- |
| (Val^8^)GLP-1 | Gault et al. (2008) [171] | Male Wistar rats/220-280 g | Aβ_25-35_ 100 nmol in 5ul i.c.v. | GLP-1 15 nmol in 5 μl i.c.v. | 1. Control 2. Aβ 3. (Val^8^)GLP-1+ Aβ | LTP measurement | (Val^8^)GLP-1 30 min prior to injection of amyloid (25-35) (100 nmol i.c.v) fully reversed the impairment of LTP induced by Aβ.  (Val^8^)GLP-1 15 min prior to simultaneously with Aβ, no such reversal was observed. |
| (Val^8^)GLP-1 | Wang et al. (2010) [217] | Male Wistar rats/200-280 g | Aβ_1-40_ (5 nmol/5 μL) i.c.v. | Administration 5μl of Val^8^GLP-1(7-36) (0.05 pmol/5 μL) i.c.v. for 3-5 min | 1. Control 2. (Val^8^)GLP-1(7-36) 3. Aβ_1-40_ 4. (Val^8^)GLP-1+ Aβ_1-40_ | In vivo hippocampal L-LTP recording & morris water maze test | Val^8^GLP-1(7-36) alone did not affect baseline synaptic transmission and L-LTP, but, pretreatment with Val^8^GLP-1(7-36) protected against Aβ_1-40_-induced impairment of L-LTP.  Val^8^GLP-1(7-36) alone did not affect learning and memory function but protected against Aβ_1-40_-induced impairment of learning and memory. |
| (Val^8^)GLP-1 | Gengler et al. (2012) [172] | APP/PS1-21 mice/9 and 18 months | Coexpression KM670/671NL mutated human amyloid precursor protein and L16 6P mutated human presenilin to model some aspects of AD development | Injection daily with (Val^8^)GLP-1 25 nmol/kg, 2.5 nmol/kg for 21 days | 1. APP/PS1-21 +saline 2. APP/PS1-21+(Val^8^)GLP-1 3. WT+saline 4. WT+(Val^8^)GLP-1 25 nmol/kg 5. WT+(Val^8^)GLP-1 2.5 nmol/kg | LTP recording in hippocampus area CA1 & staining sections for IBA-1, Aβ plaques and congophilic plaques in cortex | (Val^8^)GLP-1 rescued LTP in area CA1 of hippocampus in APP/PS1-21 mice.  The number of Aβ plaques and microglia activation in cortex increased with age but was not reduced by (Val^8^)GLP-1, however, the number of Congo red positive dose-core amyloid plaques was reduced after (Val^8^)GLP-1 treatment in 18-month-old mice. |
| (Val^8^)GLP-1 | Li et al. (2012) [149] | Male Wistar rats/250±10 g | Streptozotocin (3 mg/kg) rejection with a rate of 1 μl/min | (Val^8^)GLP-1 50 μM in 10 μl i.c.v. after 30 min for STZ administration | 1. Control 2. STZ 3. STZ+(Val^8^)GLP-1(7-36) | Water maze test & tau and p-tau levels & ultra-structural-morphometric analysis | (Val^8^)GLP-1 improved learning and memory function, and reduced total tau expression and hyperphosphorylated tau levels.  (Val^8^)GLP-1 recovered damaged cell nuclei and nucleolus. |
| (Val^8^)GLP-1 | Wang et al. (2013) [173] | Wistar rats/2-3 weeks | 100 nM Aβ | 10 nM (Val^8^)GLP-1(7-36) | 1. Control 2. Aβ_1-40_ 3. (Val^8^)GLP-1(7-36) 4. (Val^8^)GLP-1(7-36)+Aβ_1-40_ | Electrophysiological recording & confocal calcium imaging and measurement | (Val^8^)GLP-1(7-36) prevented Aβ_1-40_-induced changes in frequency of mEPSCs and mIPSCs.  (Val^8^)GLP-1(7-36) pretreatment prevented Aβ_1-40_-induced elevation of [Ca^2+^]_i_ in cultured cortical neurons. |
| GLP-1(9-36)^amide^ | Ma et al. (2012) [218] | APP/PS1 mice/10-12 months | Amyloid precursor protein/presenilin 1 mutant mice that model AD | GLP-1(9-36)^amide^ treated continuously for 2 weeks (500 ng/g/d). | 1. WT 2. APP/PS1 3. WT+GLP-1(9-36)^amide^ 4. APP/PS1+GLP-1(9-36)^amide^ | Hippocampal LTP and long-term depression (LTD) & morris water maze & visible platform task & a standard conditioned fear memory paradigm & brain levels of APP and Aβ & levels of mitochondrial superoxide & levels of hippocampal GSK3β | GLP-1(9-36)^amide^ reversed AD-related alterations in hippocampal synaptic plasticity.  GLP-1(9-36)^amide^ rescued memory deficits, but did not alter levels of APP and Aβ in APP/PS1 mice.  GLP-1(9-36)^amide^ decreased elevated levels of mitochondrial superoxide associated with AD.  GLP-1(9-36)^amide^ reversed activating dephosphorylation of hippocampal Akt and GSK3β in APP/PS1 mice. |
| GLP-1(7-36)amide | Iwai et al. (2014) [219] | Male Wistar rats/3 weeks, Male ddY mice/5 weeks | LPS 10 μg/mouse i.c.v. | GLP-1(7-36)amide 0.09-0.9 nmol/mouse i.c.v. before LPS treatment | 1. PBS 2. LPS+PBS 3. LPS+ GLP-1(7-36)amide 0.09 4. LPS+ GLP-1(7-36)amide 0.3 5. LPS+ GLP-1(7-36)amide 0.9 | Spontaneous alternation behavior via Y-maze & electrophysiological recording | GLP-1(7-36)amide significantly prevented LPS-induced impairment in spontaneous alternation performance.  GLP-1(7-36)amide significantly prevented LPS-, IL-1β-, H_2_O_2_-induced impairment in synaptic functions in CA1 region of hippocampal slices. |
| CJC-1131 | Zhang et al. (2017) [180] | Male Sprague-Dawley rats/180-220 g | Aβ_1-42_ (2.5 nmol/μl) into bilateral hippocampi. 15 min after CJC-1311 injection with an injection rate of 0.2 μl/min | CJC-1311 (2.5 nmol/μl) injection into bilateral hippocampi with a rate of 0.2 μl/min | 1. Vehicle+saline 2. Aβ_1-42_+saline 3. Vehicle+CJC-1311 4. Aβ_1-42_+CJC-1311 | Morris water maze test & hippocampal LTP recording & levels of PKA and p-PKA in hippocampus | CJC-1311 effectively prevented Aβ_1-42_-induced impairments in spatial learning and memory.  CJC-1311 reversed Aβ_1-42_-induced suppression of hippocampal LTP.  CJC-1311 increased hippocampal p-PKA levels. |
| Geniposide | Gao et al. (2014) [220] | Male Sprague Dawley (SD) rats/220-250 g | STZ 3 mg/kg i.c.v. | Geniposide 50 μM i.c.v. | 1. Control 2. Geniposide 3. STZ 4. Geniposide+STZ 5. WT+ Geniposide+STZ | Morris water maze test & p-tau, IRS-1, AKT, GSK-3β levels & structural assessment of hippocampus by TEM | Geniposide partially prevented STZ-induced learning and memory impairment via modulation of PI3K/GSK-3β signaling pathway.  Geniposide reduced STZ-induced hyperphosphorylation of tau protein, and prevented STZ-induced ultrastructure pathology. |
| Geniposide | Lv et al. (2015) [221] | Male APP/PS1 mice/6 months | MPrP-APPswe/PS1dE9 AD transgenic mice | Geniposide 25 mg/kg/d via intragastric administration | 1. WT+Vehicle 2. APP/PS1+Vehicle 3. APP/PS1+ Geniposide | Morris water maze test & immunohistochemical staining for Aβ in brain & cytokine assays & levels of RAGE, p-p42-p44 ERK1/2, ERK1/2, p-NF-κB p65, NF-κB p65, p-IκBα, IκBα and BACE1 & Aβ ELISA & field recording & whole-cell patch clamp recording from cultured hippocampal neurons | Geniposide suppressed production of proinflammatory mediators depending on RAGE-mediated signaling pathway in APP/PS1 mice.  Geniposide suppressed Aβ accumulation in APP/PS1 mice, attenuated Aβ-induced reduction of LTP in acute hippocampal slices, and attenuated Aβ-induced synaptic dysfunction in cultured hippocampal neurons.  Geniposide alleviated cognitive dysfunction in APP/PS1 mice. |
| Geniposide | Li et al. (2018) [212] | Double transgenic APPSwe/PS1 mice;  STZ-treated mice/1.5 months | Crossing of Double transgenic APPSwe/PS1 mice (AD) and STZ-treated mice (T2D);  STZ-treated mice: single intraperitoneal injection of STZ 50 mg/kg | Geniposide was added to cell cultures at a final concentration of 0, 0.1, 1.0 and 10 μM | 1. Control 2. STZ 3. APP/PS1 4. APP/PS1+STZ | TUNEL staining & cell viability assay & detection of cell apoptosis via flow cytometry & levels of IDE, cleaved caspase-3, cleaved caspase-9 and cleaved caspase-8 & quantitative real-time RT-PCR | The apoptotic cell in T2D and AD mice was dramatically increased as compared to AD mice.  Geniposide reversed effects of Aβ1-42 on cell viability and apoptosis of PC12 cells, and IDE antagonist Bac reversed the effects of geniposide.  Inhibitor of cAMP/PKA signaling pathway reversed effects of geniposide on expression of IDE. |
| Geniposide | Zhang et al. (2019) [222] | APP/PS1 double-transgenic mice/6 months | APP/PS1 mice resemble well physiopathology of human disease and develop obvious amyloid plaque deposition in brain at 6 months old | Geniposide administration intragastrically once a day for 8 weeks | 1. APP/PS1+ geniposide 2. APP/PS1 3. WT | Novel object recognition test & morris water maze test & place navigation test & spatial probe test & visible platform test & quantitation of soluble Aβ_1-40_ and Aβ_1-42_ levels by ELISA & Akt, p-Akt, mTOR, p-mTOR, 4E-BP1, P-4E-BP1 levels | Geniposide ameliorated learning and memory impairments, and improved shrinkage and necrosis neurons in APP/PS1 mice.  Geniposide reduced brain Aβ_1-40_ plaque formation and decreased soluble Aβ_1-40_ as well as Aβ_1-42_ levels in hippocampi of APP/PS1 mice.  Geniposide up-regulated brain expression of LC3-Ⅱ and Beclin 1, and reduced expression of mTOR pathway activation markers in APP/PS1 mice. |
| Exendin(5-39) | Oka et al. (2000) [223] | Male Wistar rats/170-225 g | Aβ 300 pmol/day injection into left lateral cerebroventricle | Exendin(5-39) continuous i.c.v. infusion together with Aβ(1-40) infusion | 1. Vehicle 2. Aβ 3. Aβ+Exendin(5-39) | Y-maze & recording step-through type passive avoidance response & DNA strand breaks detected through TUNEL method in hippocampal CA1 region | Co-infusion of exendin(5-39) with Aβ(1-40) prevented memory impairment and DNA fragmentation induced by Aβ in hippocampal CA1 region. |
| Exendin-4 | Perry et al. (2002) [191] | Male Fischer-344 rats/300 g | Ibotenic acid dissolved in 0.1 M PBS injection unilaterally into left lateral branch of forebrain bundle | GLP-1 2×10^-8^ M or 2×10^-9^ M exendin-4 injection into right lateral ventricle at a rate of 0.25 μl/h over 14 days | 1. Vehicle 2. Exendin-4 3. GLP-1 | Intracellular cAMP determination & cell survival & immunohistochemical staining for ChAT and GFAP | GLP-1 and exendin-4 protected culture hippocampal neurons against cell death induced by glutamate.  GLP-1 and exendin-4 attenuated Ibotenic acid-induced cholinergic marker deficit in adult rats. |
| Exendin-4 | Perry et al. (2003) [152] | Male db/db mice | Type 2 diabetes mice | GLP-1 3.3 ng, Exendin-4 0.2 ng, NGF 2 μg into lateral ventricles at 0.25 μl/min over 4 min | 1. Control 2. GLP-1 3. Exendin-4 4. NGF | Aβ assay & lactate dehydrogenase assay | GLP-1 and exendin-4 could reduce levels of Aβ in brain *in vivo* and reduce levels of APP in cultured neuronal cells.  GLP-1 and exendin-4 protected cultured hippocampal neurons against death induced by Aβ and iron. |
| Exendin-4 | Li et al. (2010) [151] | 3×Tg-AD mice/11-12.5 months | STZ (0.1 ml volume) i.p. for 5 consecutive days at a dose of 50 mg STZ/kg body weight | Subcutaneously implanted micro-osmotic pumps at a rate of 3.5 pM/kg/min.  Exendin-4 3 days after the final STZ dose to 16-week. | 1. Control 2. Low dose STZ 3. Exendin-4 only 4. STZ + exendin-4 | AβPP, Aβ and tau levels in right hippocampus homogenates & intraperitoneal glucose tolerance test (IPGTT) & plasma insulin & hemoglobin A1c (HbA1c) levels | Exendin-4 ameliorated STZ-induced diabetes in 3×Tg-AD mice.  Exendin-4 ameliorated levels of AβPP and Aβ in 3×Tg-AD mice brain of STZ-induced diabetes.  Neither STZ-induced diabetes nor exendin-4 treatment significantly affected brain levels of total tau in 3×Tg-AD mice |
| Exendin-4 | Bomfim et al. (2012) [224] | APP/PS1 mice | AD Tg mouse model | Exendin-4 25 nmol/kg intraperitoneal injection (i.p.) injection twice daily for 3 weeks | 1. WT 2. Saline 3. Exendin-4 | Immunocytochemical staining for AβO, IRS-2, IRS-1 and p-IRS-1 & levels of p-IRS-1, IRS-1, IRS-2, p-JNK, JNK, TNF-α, TNFR1, GLP-1R and cyclophilin B & cellar live imaging & morris water maze task & immunohistochemical staining for Aβ plaques & ELISA for total soluble Aβ levels | Exendin-4 decreased levels of hippocampal IRS-1pSer and activated JNK.  Exendin-4 improved behavioral measures of cognition in APP/PS1 mice.  Exendin-4 prevented Aβ oligomers-induced impaired axonal transport.  Effects of exendin-4 were reversed by GLP-1 antagonist exendin 9-39.  Exendin-4 decreased levels of soluble Aβ and amyloid plaque load. |
| Exendin-4 | Chen et al. (2012) [182] | Male Wistar rats/210-260 g | STZ (3 mg/kg, 6 μl each side for a rat) i.c.v. | Exendin-4 (10 μg/kg) subcutaneous (s.c.) injection twice daily for 14 days | 1. Control 2. STZ+exendin-4 3. STZ+Vh | Glucose measurements & morris water maze & histopathological examination & total GSK-3β, ser9-phosphorylated GSK-3β, tyr216-phosphorylated GSK-3β, ser473-phosphorylated Akt, total Akt, thr205-phosphorylated tau, tau, ser396-phosphorylated tau, thr181-phosphorylated tau levels | Exendin-4 protected PC12 cells against damage induced by high glucose through PI3K pathway.  Exendin-4 ameliorated oxidative stress-induced injury in PC12 cells.  Exendin-4 improved learning and memory performance and protected hippocampal neurons against degeneration in STZ-treatment rats.  Exendin-4 reversed STZ-induced tau hyperphosphorylation through downregulation of GSK-3β activity. |
| Exendin-4 | Ohtake et al. (2014) [178] | Male CD1 mice/9-11 weeks | Human soluble Aβ oligomer i.c.v. | Exendin-4 (0.2 mg/kg at each time) subcutaneous injection five times at 3-h intervals | 1. Control 2. Exendin-4 | AMPA-GluR1, ADAM10, CREB, p-CREB, BDNF, PSD-95, APP, NMDA-NR1, AMPA-GluR2, p-mTOR and mTOR levels | Exendin-4 increased CREB phosphorylation and BDNF expression in mice neocortex.  Exendin-4 promoted membrane insertion of GluR1, but not of GluR2 and NR1, in mouse neocortex.  Exendin-4 increased α- and β-CTF levels and promoted ADAM10 membrane insertion.  Exendin-4 upregulated protein levels of AMPA receptor GluR1 subunit and ADAM in plasma membrane fraction of mouse neocortex following Aβ oligomer i.c.v. |
| Exendin-4 | An et al. (2015) [28] | ICR mice/6 weeks/ 30-33g | Advanced glycation end product (AGEs) (1 μg/kg) injection into the tail vein every 2 days | Exendin-4 25 nm/kg injection into abdominal cavity twice daily | 1. Control 2. AGEs 3. AGEs+exendin-4 | Levels of tau phosphorylation & intracellular ROS measurement & mitochondrial membrane potential measurement & mitochondrial antioxidant enzyme activities measurement in hippocampal & Total GSK3β, ser9-p-GSK3β, PP2Ac, p-PP2A levels | GLP-1/exendin-4 reduced tau hyperphosphorylation, ROS damage, mitochondrial damage, antioxidant enzyme activities in hippocampal mitochondria.  GLP-1/exendin-4 promoted mitochondrial biogenesis by activation of PGC-1α and regulated activity of GSK3β. |
| Exendin-4 | Wang et al. (2015) [205] | Male Sprague-Dawley (SD) rats/220-260 g | Aβ_1-42_ 0.625 nmol intrahippocampal injection | Exendin-4 0.2 nmol intrahippocampal injection | 1. Control 2. Aβ_1-42_ 3. Exendin-4+Aβ_1-42_ 4. Exendin-4 | Examination of LTP in hippocampal CA1 region & immunohistochemical staining for p-CaMKⅡα expression & Confocal calcium imaging and measurements | Exendin-4 partially antagonized Aβ_1-42_-induced impairment of LTP.  Exendin-4 antagonized Aβ_1-42_-induced calcium overload by regulating calcium homeostasis, and antagonized reduced expression of CaMKⅡα induced by Aβ_1-42_ in hippocampus of rats. |
| Exendin-4 | Xu et al. (2015) [155] | Male Sprague-Dawley rats/200-250 g | After 12 weeks of high fat diet, STZ i.p. once with a dose of 60 mg/kg | Exendin-4 3.2 μg/kg i.p. for 28 days | 1. Control 2. T2D 3. T2D+Exendin-4 | Blood glucose & insulin and HOMA-insulin resistance & levels of phosphorylated tau, Tau5, Tau1, t-AKT, p-AKT, GSK-3β and p- GSK-3β | Exendin-4 ameliorated hyperglycemia and insulin resistance of T2D rats.  Exendin-4 reduced hyperphosphorylated tau in hippocampus.  Exendin-4 restored AKT and GSK-3β activity in brain. |
| Exendin-4 | Wang et al. (2016) [85] | Sprague-Dawley rats/220-260 g | Intrahippocampal injection Aβ_1-42_ 0.625 nmol | Intrahippocampal injection exendin-4 0.2 nmol | 1. Control 2. Aβ_1-42_ 3. Exendin-4+ Aβ_1-42_ 4. Exendin-4 | Morris water maze test & hippocampal CA1 region LTP recording & cAMP calculating & p-CREB levels | Exendin-4 mitigated Aβ_1-42_-induced abnormal behavior.  Exendin-4 prevented Aβ_1-42_-induced impairment of LTP.  Exendin-4 antagonized Aβ_1-42_-induced decrease of cAMP and p-CREB in rat hippocampus. |
| Exendin-4 | Wang et al. (2016) [225] | Male C57BL/6 mice/8-10 weeks/20±2 g | Intrahippocampal injection of 15 nmol Aβ_31-35_ | Intranasal/intrahippocampal administration of 0.5 nmol Exendin-4 | 1. Control 2. Aβ_31-35_ 3. Exendin-4 (Nasal) + Aβ_31-35_ 4. Exendin-4 (Hippocampus) + Aβ_31-35_ 5. Exendin-4 (Nasal) 6. Exendin-4 (Hippocampus) | Wheel-running activity & morris water maze training & ELISA for exendin-4 levels in hippocampus | Intranasally and intrahippocampally administered exendin-4 ameliorated Aβ_31-35_-induced disruption of circadian rhythm and impairment of learning and memory. |
| Exendin-4 | Yang et al. (2016) [154] | Sprague-Dawley rats/250-275 g | After 12 weeks of high fat diet, STZ i.p. once with a dose of 30-35 mg/kg | Exendin-4 3.2 μg/kg i.p. | 1. Veh/veh 2. Veh/wort 3. Exendin-4/veh 4. Exendin-4/wort | Insulin, p-IRS1, t-IRS1, P-GSK3β, t- GSK3β, p-AKT, t-AKT, t-tau and p-tau levels | Exendin-4 decreased hyperphosphorylation of tau, and enhanced insulin signaling via increasing insulin levels in brains of T2D rats. |
| Exendin-4 | Jia et al. (2016) [226] | Male Sprague-Dawley rats/230-250 g | Aβ_1-42_ 5 nmol i.c.v. | Exendin-4 0.02, 0.2, 2 nmol i.c.v. | 1. Control 2. Aβ_1-42_ 3. Exendin-4 0.02 nmol 4. Exendin-4 0.2 nmol 5. Exendin-4 2 nmol 6. Exendin-4+ Aβ_1-42_ | Morris water maze test & levels of Bax, Bcl2 and caspase-3 | Exendin-4 prevented Aβ_1-42_-induced impairment of spatial learning and memory in a dose-dependent manner.  Exendin-4 effectively prevented Aβ_1-42_-induced change in levels of Bax/Bcl2 and caspase-3 in hippocampus of rats. |
| Exendin-4 | Wang et al. (2018) [227] | Male APP/PS1 mice/6 months/30-45 g | Amyloid precursor protein/presenilin 1 mutant mice that model AD | Exendin-4 (25 nmol/kg) injection twice daily | 1. WT 2. APP/PS1 3. APP-PS1+exendin-4 | Morris water maze & GnT-Ⅲ, GnT-Ⅴ and p-tau levels in brain, | Exendin-4 improved memory deficits in APP/PS1 mice.  Exendin-4 reduced levels of accumulated Aβ and hyperphosphorylated tau, and GnT-Ⅲ levels in APP/PS1 mice hippocampus and cortex. |
| Exedin-4 | Garabadu et al. (2019) [189] | Male wistar albino rats/6-8 weeks/180±20 g | Aβ (1 μg/μl, 5μl/animal; 1.0 μl/min) i.c.v. | Exendin-4 5μg/kg i.p. | 1. Control 2. Sham 3. Aβ 4. Aβ+Exendin-4 5. Aβ+Exendin-4+LY294002 6. Aβ+Donepezil | Morris water maze test & Y-maze test, assay of activity of ChAT & analysis of Ach level & estimation of activity AChE in different brain regions & estimation of mitochondrial function & evaluation of MMP in discrete brain regions & estimation of mitochondrial bioenergetics & estimation of mitochondrial respiratory complex-Ⅰ, Ⅱ, Ⅳ and Ⅴ activity & estimation of cytosolic Aβ, Akt and pAkt levels | Exendin-4 ameliorated Aβ_1-42_-induced memory deficits and accumulation of Aβ.  Exendin-4 improved cholinergic activity, and attenuated Aβ_1-42_-induced decrease in mitochondrial function as well as integrity.  Exendin-4 ameliorated Aβ_1-42_-induced decrease in mitochondrial RCR and ADP/O in rat.  Exendin-4 ameliorated Aβ_1-42_-induced decrease of pAkt and changes in mitochondrial complex enzyme activities. |
| Exendin-4 | King et al. (2020) [156] | Female TAPP mice | Mutant human APP and human tau (Tg(APPSWE)2576KhaTg(Prnp-MAPT*P301L)JNPL3HImc) | Exendin-4 10 μg/kg s.c. twice per day for 6 weeks | 1. WT 2. WT-STZ 3. WT-HFD 4. TAPP 5. TAPP-STZ 6. TAPP-HFD | Glucose tolerance test & barnes circular maze task & plasma insulin, adiponectin and TG levels analysis & levels of p-tau, tau and Aβ | Levels of phosphorylated tau were increased in brain of WT-STZ and TAPP-STZ mice but not in brain of WT-HFD and TAPP-HFD.  Exendin-4 improved learning ability, and reduced Aβ and phosphorylated tau expression. |
| NLY01 (engineered exendin-4) | Park et al. (2021) [228] | 5×FAD mice/3 months and 3×Tg-AD mice/7 months | 5×FAD (B6SJL-Tg, (APPSwFILon, PSEN1*M146L*L286V)6799Vas/Mmjax), 3×Tg-AD (B6; 129-Psen1^tm1Mpm^Tg (APPSwe, tauP301L)1Lfa/Mmjax) | Administration NLY01 1 or 10 mg/kg by subcutaneous injection twice a week for 4 months (5×FAD) or 5 months (3×Tg-AD) | 1. WT+PBS 2. WT+NLY01-10 3. 5×FAD+PBS 4. 5×FAD+NLY01-1 5. 5×FAD+NLY01-10 | Morris water maze & passive avoidance test & Y-maze, exendin-4 detection & glucose level measurement & GLP-1R, Iba-1, GFAP, MBP, TBR1, CTIP, SATB2, MAP2, 4G8 immunostaining & GLP-1R, GFAP, Iba-1, Tuj1, C3, MAP2, BDNF, Bcl-2, PSD95 levels & cytokine analysis & cell death and viability analysis | NLY01 ameliorated cognitive deficits in 5×FAD mice.  NLY01 attenuated Aβ_1-42_ induced microglia activation through GLP-1R.  NLY01 prevented reactive astrocyte conversion induced by Aβ_1-42_ activated microglia.  NLY01 rescued neuronal cell death by prevention of reactive astrocyte conversion, and rescued AD-related pathology in 3×Tg-AD mice. |
| Liraglutide | McClean et al. (2011) [175] | APP/PS1 mice/7 months | APP_swe_/PS1_ΔE9_ transgenic mice | Liraglutide 25 nm/kg daily for 8 weeks | 1. WT+Saline 2. WT+Liraglutide 3. APP/PS1+Saline 4. APP/PS1+Liraglutide | Object recognition task & morris water maze task & reversal morris water maze task & analysis of transport across BBB & electrophysiological recording in hippocampus area CA1 & immunohistochemical staining for Iba 1, Aβ plaque, congophilic plaques, synaptophysin and doublecortin & ELISA for total soluble β-amyloid and APP levels | Liraglutide improved object recognition, and spatial memory in water maze.  Liraglutide enhanced induction and maintenance of LTP and PPF in APP/PS1 and WT mice.  Liraglutide dramatically reduced histological hallmarks (β-amyloid plaque formation, number of dense-core Congo red plaques, inflammatory response) of AD in APP/PS1 mice.  Liraglutide increased number of young neurons and synaptophysin levels in APP/PS1 mice.  Liraglutide significantly reduced soluble Aβ oligomer and total APP levels. |
| Liraglutide | Yang et al. (2013) [229] | Male Wistar rats/10-12 weeks | STZ 200 mg/kg i.p. after feeding with high fat, high glucose and high protein diet for 12-16 weeks | Liraglutide 0.2 mg/kg s.c. twice a day for up to four weeks after three days STZ injection | 1. CTL 2. T2D+Saline 3. T2D+Liraglutide | Glucose, insulin, GLP-1 and HOMA-IR measurements & t-tau and p-tau levels & immunohistochemical staining for p-tau | Liraglutide ameliorated hyperglycemia and insulin resistance of type 2 diabetic rats.  Liraglutide reduced tau phosphorylation, and normalized brain AKT as well as GSK-3β activity in brains of type 2 diabetic rats. |
| Liraglutide | Han et al. (2013) [177] | Male Sprague-Dawley (SD) rats/230-250 g | Aβ_25-35_ 4 nmol injection into bilateral hippocampus | Liraglutide 0.05-5 nmol injection into bilateral hippocampus with an injection rate of 0.2 μL/min.  Liraglutide 25 nmol/kg i.p. injection for cAMP assay. | 1. Control 2. Aβ 3. 0.05 nmol Liraglutide+ Aβ 4. 0.5 nmol Liraglutide+ Aβ 5. 5 nmol Liraglutide+ Aβ | Morris water maze task & in vivo hippocampal L-LTP recording & cAMP assay | Liraglutide alone did not affect normal cognitive behavior but dose-dependently prevented against Aβ_25-35_-induced impairment of spatial learning and memory.  Liraglutide partly and dose-dependently prevented Aβ_25-35_-induced depression of hippocampal L-LTP, and upregulated intracellular cAMP level. |
| Liraglutide | Long-Smith et al. (2013) [214] | APP/PS1 mice/7 months | APP_swe_/PS1_dE9_ transgenic mice | Liraglutide 25 nM/kg injection for 8 weeks | 1. WT 2. APP/PS1+Saline 3. APP/PS1+Liraglutide | Immunofluorescence for Aβ, IRβ, GFAP and Iba-1 | Liraglutide significantly reduced Aβ plaque levels, changes in IR localization, heightened IRS-1 pS^616^ levels, levels of astrocytic activation, levels of activated microglia and microglial number in APP/PS1 mice. |
| Liraglutide | Parthsarathy et al. (2013) [168] | APP/PS1 mice/3,6,12,15 months | APPswe/PS1 dE9 transgenic mice | Liraglutide 25 nmol/kg i.p. for 7 days (acute) and 37 days (chronic) | 1. WT+Saline 2. WT+Liraglutide 3. APP/PS1+Saline 4. APP/PS1+Liraglutide | BrdU and Doublecortin staining & Ki67 staining & Double immunofluorescence staining | Acute liraglutide increased BrdU positive cells and neuroblast differentiation.  Chronic liraglutide increase neuroblast differentiation and neuronal differentiation. |
| Liraglutide | Xiong et al. (2013) [216] | Male Kunming mice/3 months/38.66±1.80 g | STZ 3 mg/kg i.c.v. injection into right lateral ventricle | Liraglutide 300 μg/kg s.c. injection for 30 days | 1. Control 2. STZ 3. STZ+Liraglutide | Levels of SMI31, RL2, R61d, PHF1, tau-5, p-tau, JNK/SAPKs, p-JNK/SAPKs, ERK and p-ERK & immunohistochemical staining for NF and tau phosphorylation & microtubule binding assay & morris water maze | Weight and blood sugar were not different among three groups.  Liraglutide decreased hyperphosphorylation of NFs and hyperphosphorylated tau, and increased glycosylation in brain of STZ mice.  Liraglutide improved microtubule binding tau impaired by STZ, and ameliorated ERK and INK signaling pathways in STZ mice brain.  Liraglutide protected STZ mice from neurodegeneration, and improved learning and memory impairment induced by STZ. |
| Liraglutide | McClean et al. (2014) [158] | APP_swe_/PS1_ΔE9_ mice/14 months | A model of AD | Liraglutide 25 nm/kg i.p. once daily for 8 weeks | 1. WT+Saline 2. WT+Liraglutide 3. APP/PS1+Saline 4. APP/PS1+Liralutide | Object recognition task & morris water maze task & LTP recording in hippocampus area CA1 & Iba-1, Doublecortin and Aβ histology staining & ELISA for total soluble Aβ and APP levels | Liraglutide restored object recognition memory, and reduced Aβ load, soluble amyloid oligomer levels and inflammation in aged APP/PS1 mice.  Liraglutide enhanced in vivo LTP in aged APP/PS1 and WT mice.  Liraglutide increased synaptophysin and IDE levels in aged APP/PS1 mice. |
| Liraglutide | McClean et al. (2015) [143] | APP/PS1 mice/2 months | APP_swe_/PS1_ΔE9_ transgenic mice | Liraglutide 25 nm/kg i.p. once daily for 8 months | 1. WT+Saline 2. APP/PS1+Saline 3. APP/PS1+Liraglutide | Blood glucose measurement & object recognition task & morris water maze task & LTP recording in hippocampus area CA1 & immunohistochemical staining for Iba-1, Aβ, synaptophysin and DCX | Liraglutide maintained recognition memory in APP/PS1 mice.  Liraglutide improved morris water maze performance, and enhanced LTP in APP/PS1 mice.  Liraglutide dramatically reduced histological hallmarks of AD in area CA1 of APP/PS1 mice.  Liraglutide increased synaptophysin levels in APP/PS1 mice. |
| Liraglutide | Ma et al. (2015) [161] | Male db/db mice/2-2.5 weeks | BKS.Cg-Dock7 ^m^ +/+ Lepr ^db^/JNju | Subcutaneous injection liraglutide 0.1 mg/kg or insulin 0.67 U/kg each day for 2-8 weeks | 1. Control 2. Liraglutide 3. Insulin | Measurements of blood glucose and plasma insulin & measurements of CSF insulin & levels of t-tau, p-tau, p-Akt, t-Akt, p-GSK-3β and GSK-3β | Liraglutide prevented weight gain, reduced insulin resistance, and by 8 weeks restored CSF insulin in young db/db mice.  Liraglutide prevented age-dependent increase in tau phosphorylation in hippocampal formation of db/db mice.  Liraglutide prevented dysregulation of Akt and GSK-3β in brain of db/db mice. |
| Liraglutide | Hansen et al. (2015) [169] | SAMP8 mice/6 months/28.0±0.6 g | Senescence-accelerated mouse prone 8 (SAMP 8) mice, a model of age-related sporadic AD not dominated by amyloid plaques | Liraglutide 100 or 500 μg/kg/day s.c. once daily for 4 months | 1. SAMP8 50% back-crossed 2. SAMP8 4 months old 3. SAMP8 vehicle 4. SAMP8 liraglutide 100 μg/kg 5. SAMP8 liraglutide 500 μg/kg | Active avoidance T-maze & novel object recognition task & immunohistochemical staining for Aβ and phosphorylated tau & stereological quantification of hippocampal CA1 pyramidal neuron numbers | Liraglutide did not affect body weight and blood glucose.  Liraglutide improved memory retention in an active-avoidance T-maze task, but did not affect novel object recognition.  Liraglutide preserved hippocampal CA1 pyramidal neuron numbers in SAMP8 mice. |
| Liraglutide | Spolcova et al. (2015) [230] | NMRI mice/6 months | L-glutamic acid sodium salt hydrate administration s.c. (MSG-induced obesity) | Palm-PrRP31 5 mg/kg or liraglutide 0.2 mg/kg s.c. injection twice per day for 2 weeks | 1. MSG-obese+Saline 2. MSG-obese+Palm-PrRP31 3. MSG-obese+Liraglutide | Glucose tolerance test & determination of plasma insulin and leptin concentrations via ELISA & levels of p-GSK-3β, t-GSK-3β, p-Tau, t-Tau, p-PDK1, t-PDK1, p-Akt, t-Akt, p-MAPK/ERK1/2, t-MAPK/ERK1/2, p-JNK and t-JNK & immunohistochemical staining for phosphorylated tau & prolactin releasing hormone receptor (PrRP-R) immunohistochemistry | Palm-PrRP31 and liraglutide attenuated food intake but did not affect body weight, fasting glucose and insulin levels in MSG-obese mice.  Palm-PrRP31 and liraglutide ameliorated insulin signaling, and attenuated activity of tau kinases and tau hyperphosphorylation in hippocampus of MSG-obese mice. |
| Liraglutide | Qi et al. (2016) [164] | Male C57/BL6/8 weeks | Aβ_1-42_ 410 pmol/5 μl with a rate of 0.2 μl/min injection into lateral ventricle | Liraglutide 25 nmol/kg s.c. once daily for 8 weeks | 1. Control 2. Liraglutide 3. Aβ 4. Aβ+Liraglutide | Morris water maze test & Y-maze test & Glucose, insulin, glucagon and GLP-1 receptor measurements & observation of ultrastructure of cells in hippocampal CA1 region via TEM & immunohistochemical staining for p-tau & levels of p-tau, tau, p-Akt, Akt, p-GSK-3β and GSK-3β | Liraglutide increased expression of GLP-1R in hippocampus.  Liraglutide improved cognitive performance in Y maze and morris water maze test.  Liraglutide alleviated chemical synapses changes and tau hyperphosphorylation induced by Aβ_1-42_.  Liraglutide normalized brain AKT andGSK-3β activity. |
| Liraglutide | Hansen et al. (2016) [231] | Female transgenic hAPP_Lon_/PS1_A246E_ mice/5 months and female hAPP_Swe_/hPS1_ΔE9_ mice/7 months | Transgenic hAPP_Lon_/PS1_A246E_ mice expressing both human clinical ‘London’ mutant V717I variant of amyloid precursor protein and a clinical mutant variant of human presenilin 1.  Transgenic hAPP_Swe_/hPS1_ΔE9_ mice expressing both human clinical ‘Swedish’ mutant K595N/M596L variant of amyloid precursor protein and an exon-9-deleted clinical mutant variant of human presenilin 1. | Liraglutide 100 or 500 ng/kg/day s.c.  hAPP_Lon_/PS1_A246E_ mice s.c. liraglutide 100 or 500 ng/kg/day for 3 months.  hAPP_Swe_/hPS1_ΔE9_ mice s.c. liraglutide 500 ng/kg/day for 5 months. | 1. WT+Vehicle 2. hAPP_Lon_/PS1_A246E_+Vehicle 3. hAPP_Lon_/PS1_A246E_+Liraglutide-100 4. hAPP_Lon_/PS1_A246E_+Liraglutide-500 5. hAPP_Swe_/hPS1_ΔE9_+Vehicle 6. hAPP_Swe_/hPS1_ΔE9_+Liraglutide | Morris water maze task & novel object recognition task & active avoidance -maze task & assessment of Aβ plaque load and brain volume | Liraglutide had no effect on behavioral tasks in hAPP_Lon_/PS1_A246E_ and hAPP_Swe_/hPS1_ΔE9_ mice.  Liraglutide had no effect on Aβ plaque levels in hAPP_Lon_/PS1_A246E_ and hAPP_Swe_/hPS1_ΔE9_ mice. |
| Liraglutide | Hansen et al. (2016) [163] | Female transgenic tauopathy mice/3 months | Transgenic mutant tau (hTauP301L) mouse tauopathy model, developing age-dependent pathology-specific neuronal tau phosphorylation and neurofibrillary tangle formation | Liraglutide 500 μg/kg/day s.c. once daily for a total duration of 22 weeks;  Initial dose of 25 μg/kg/day and then increased through daily increments (50-100-150-200-250-300-500 μg/kg/day) until reaching target dose on treatment day 7 | 1. Control 2. hTauP301L+vehicle 3. hTauP301L+liraglutide | Clasping behavior & analysis of total tau levels in cerebrospinal fluid & assessment of neuronal p-tau load and brain volume | Liraglutide reduced severity of clasping behavior and improved survival rate in hTauP301L mice.  Liraglutide reduced phosphorylated tau burden in hTauP301L mice. |
| Liraglutide | Qi et al. (2017) [211] | Male C57BL/6J/9 weeks | Methylglyoxal (MG) (0.35, 0.7 or 1.4 μmol), Aβ_1-42_ (410 pmol) in a volume of 3 μl i.c.v. | Liraglutide 25 nmol/kg s.c. once daily for 8 weeks | 1. Control 2. Liraglutide 3. MG 4. MG+Liraglutide | Morris water maze & Glucose, insulin, glucagon and GLP-1R measurements & observation of synaptic structure via TEM & tau, p-tau, Akt, p-Akt, GSK-3β, p-GSK-3β, pro-caspase-3 and cleaved caspase-3 levels | Liraglutide alone did not affect normal cognitive behavior but prevented MG-induced impairment of spatial learning and memory.  Liraglutide increased hippocampal GLP-1R expression, and protected against synaptic and cellular ultrastructural changes induced by MG.  Liraglutide reduced MG-induced activated caspase-3 expression and tau hyperphosphorylation, and activated GSK-3β signaling pathway. |
| Liraglutide | Chen et al. (2017) [232] | 3×Tg mice/7 months | APP/PS1/Tau triple transgenic AD model mice | Liraglutide 300 μg/kg s.c. once daily for 8 weeks | 1. WT 2. WT+Liraglutide 3. 3×Tg 4. 3×Tg+Liraglutide | Body weight and blood glucose levels measurement & p-tau, tau5, pERK1, pERK2, ERK1, ERK2, pJNK1, pJNK2, JNK1 and JNK2 levels in brain & detection degenerated neurons via Fluoro-Jade B labeling & morris water maze test | Liraglutide improved learning and memory performance, and attenuated hyperphosphorylation of tau as well as NFs in brain of 3×Tg mice.  Liraglutide improved JNK and ERK signaling, and prevented neurodegeneration in brain of 3×Tg mice. |
| Liraglutide | Batista et al. (2018) [176] | Male Swiss mice/2.5-3 months | AβOs 10 pmol injection into lateral ventricle;  AβOs 10-100 μg injection per day every 3 days for up to 24 days | Liraglutide 25 nmol/kg i.p. daily for 7 days;  Liraglutide 0.006 mg/kg for first week and 0.012 mg/kg until end of AβO injection | 1. Vehicle 2. AβOs 3. Vehicle+Saline 4. Liraglutide+Saline 5. Liraglutide+ AβOs 6. Exendin-9-39+Liraglutide+ AβOs 7. FSK+ AβOs 8. FSK 9. PKI+Lirgalutide+ AβOs 10. 8-Br-Camp+AβOs 11. Insulin+ AβOs 12. Sham | Immunocytochemical staining for synaptophysin and PSD-95 & ELISA for PKA and insulin & synaptic image analysis & ultrastructural analysis of synapses via electron microscopy, | Liraglutide protected against AβOs-induced synapse damage via activation of cAMP/PKA signaling in hippocampal neurons.  Liraglutide regulated PKA activity and protected against memory impairment induced by AβOs in mice.  Liraglutide prevented AβOs-induced decrease in IRs in neuronal cultures and mice.  Liraglutide alleviated AβOs-induced synapse loss and tau hyperphosphorylation in NHPs. |
| Liraglutide | Zhang et al. (2019) [162] | Male Sprague-Dawley rats/250±20 g | Homocysteinemia 400 μg/kg via vena caudalis per day for 14 days | Liraglutide 150 μg/kg, 300μg/kg, or 450 μg/kg s.c. per 12 h for 14 days | 1. Control 2. Hyperhomocysteinemia 3. Hyperhomocysteinemia+150 μg/kg liraglutide 4. Hyperhomocysteinemia+300 μg/kg liraglutide 5. Hyperhomocysteinemia+450 μg/kg liraglutide | Morris water maze test & oral glucose tolerance test (OGTT) & Golgi-Cox staining & plasma hyperhomocysteinemia, insulin and Aβ analysis via ELISA & pT231 immunohistochemical staining & PSD95, Synapsin Ⅰ, GluA1, GluA2, GluN2A, GluN2B, p-tau, Tau5, pPP2Ac, Dm PP2Ac, PP2Ac, p-APP, APP, BACE1, ADAM10, PSEN1, GLP-1R, p-IRS, IRS and PTP1B levels | Liraglutide ameliorated Hyperhomocysteinemia-induced memory deficits and tau phosphorylation as well as Aβ overproduction.  Liraglutide rescued Hyperhomocysteinemia-inhibited spine formation and expression of synapse-associated proteins in hippocampus.  Liraglutide activated PP2A and restored Hyperhomocysteinemia-induced impairment of insulin sensitivity. |
| Liraglutide | Holubova et al. (2019) [159] | Male APP/PS1 mice | APPswe/PSEN1dE9 mice | Liraglutide 0.2 mg/kg s.c. or palm^11^-PrRP31 5mg/kg s.c. for 2 months | 1. WT+Saline 2. APP/PS1+Saline 3. APP/PS1+Liraglutide 4. APP/PS1+ palm^11^-PrRP31 | Determination of hormonal and biochemical parameters & Aβ, Iba1 and GFAP immunohistochemistry staining & caspase 3 levels | Liraglutide and palm^11^-PrRP31 reduced Aβ plaque load and neuroinflammation in APP/PS1 mice.  Liraglutide and palm^11^-PrRP31 reduced hippocampal levels of caspase 3 and tau hyperphosphorylation in APP/PS1 mice. |
| Liraglutide | Duarte et al. (2020) [146] | Female 3×Tg-AD mice/10 months | A genetic model for AD developing an age-related progressive neuropathological phenotype | Liraglutide 0.2 mg/kg s.c. for 28 days | 1. WT 2. 3×Tg-AD 3. 3×Tg-AD+Liraglutide | Routine biochemical analysis & evaluation of AD pathological hallmarks (Aβ_1-42_, Aβ_1-40_ and p-tau) & open field behavior test & Y-maze behavior test & morris water maze test & evaluation of inflammation markers and brain cortical hormones’ levels & assessment of brain cortical PKA activity and brain cortical glucose levels & determination of brain markers for glycolysis and pentose phosphate pathway & evaluation of oxidative/nitrosative stress markers & GLUT1, GLUT4, Fis 1 and OPA1 levels | Liraglutide partially normalized brain levels of estradiol and GLP-1-related signaling, and partially rescued brain oxidative/nitrosative stress markers in female mice with early AD-like pathology.  Liraglutide promoted brain glucose metabolism via oxidative branch of pentose phosphate pathway in female mice with early AD-like pathology.  Liraglutide partially attenuated altered mitochondrial fission/fusion proteins in female mice with early AD-like pathology. |
| Liraglutide | Carranza-Naval et al. (2021) [210] | AD-T2D mice/6 weeks | Cross-breeding APPswe/PS1dE9 with db/db mice | Liraglutide s.c. with an initial dose of 25 μg/kg/day, increasing to 50. 100, 150, 200, 300, and 500 μg/kg/day daily during first week from day seven, 500 μg/kg/day for 20 weeks | 1. Control 2. APP/PS1 3. db/db 4. APP/PS1xdb/db | Metabolic determination & morris water maze & actimetry and new object discrimination task & rotarod & cresyl violet staining & Prussian blue staining for quantifying hemorrhage burden in cortex and hippocampus & NeuN staining & axonal immunostaining & Aβ and microglia immunostaining & ELISA for Aβ40, Aβ42 and Aβ aggregates & t-tau and p-tau levels | Liraglutide reduced metabolic alterations in T2D and AD-T2D mice.  Liraglutide improved cognitive impairment, limited brain atrophy as well as neuronal loss, and reduced neuronal curvature in AD-T2D mice.  Liraglutide reduced Aβ and tau pathology.  Liraglutide reduced spontaneous bleeding and microglia activation in AD-T2D mice. |
| Liraglutide | Paladugu et al. (2021) [147] | 5×FAD and wild type mice/3 months | STZ 3 mg/kg i.c.v. | Liraglutide 25 nM/kg i.p. once a day for 30 days | 1. WT+Vehicle 2. WT+STZ 3. WT+STZ+Liraglutide 4. WT+Liraglutide 5. 5×FAD+Vehicle 6. 5×FAD+STZ 7. 5×FAD+STZ+Liraglutide 8. 5×FAD+Liraglutide | Open field task & novel object recognition & passive avoidance & GFAP, Iba-1, Aβ levels via immunohistochemical analysis & Aβ, IDE, p-AKT, AKT, GSK3β, P-GSK3β and p-IR levels | Liraglutide reduced astrocytes and microglia activity in cortical and hippocampal CA1 and CA3 regions.  Liraglutide reduced amount of Aβ levels in cortical and hippocampal regions of 5×FAD mice.  Liraglutide increased levels of IDE in cortical and hippocampal regions, and increased phosphorylated insulin receptor in cortical regions of WT and 5×FAD mice.  Liraglutide increased levels of pGSK3β in cortical region of both SAD and 5×FAD mice and only in hippocampal region of 5×FAD mice. |
| Liraglutide | Abd el-Rady et al. (2021) [26] | Male Wistar albino rats/250±20 g | Oral administration of a freshly prepared solution of AlCl_3_ at a dose of 100 mg/kg for 6 weeks | Liraglutide 300 μg/kg per day subcutaneously for 6 weeks after oral AlCl_3_ | 1. Control 2. Liraglutide 3. AlCl_3_ 4. Liraglutide+ AlCl_3_ | Eight radial arm maze & serum blood glucose & proinflammatory cytokine & oxidative stress markers & hippocampal tissue homogenate neurotransmitters & APP immunostaining & NeuN immunostaining | Liraglutide prevented impairment of learning and memory via significant reduction of serum tumor factor (TNF-α), interferon-γ (INF-γ) and malondialdehyde (MDA), and increase of superoxide dismutase (SOD), dopamine, adrenaline and noradrenaline.  Liraglutide improved hippocampal histological features of AlCl_3_ administrated rats and decreased percentage of neuronal loss. |
| Liraglutide | Xie et al. (2021) [188] | 5×FAD mice/6 months | 5×FAD transgenic mice | Subcutaneous administration 25 nmol/kg/qd liraglutide for 8 weeks | 1. WT 2. 5×FAD 3. 5×FAD+liraglutide | Nissl staining & cortex cAMP contents & brain ATP levels & ROS production & p-DRP1, t-DRP1, MFN2, OPA1 levels & mitochondrial morphology analysis & mitochondrial ROS production & measurement of cellular oxygen consumption rate & measurement of mitochondrial membrane potential | Liraglutide ameliorated mitochondrial dysfunction, prevented neuron loss, and activated cAMP/PKA pathway in brain of 5×FAD mice.  GLP-1 prevented mitochondrial fragmentation, ameliorated mitochondrial dysfunction, and promoted cell survival, and improved neuronal supportive ability in Aβ-treated astrocytes via cAMP/PKA pathway |
| Liraglutide | Zheng et al. (2021) [208] | Male 5×FAD mice/4 months | 5×FAD transgenic mice | Subcutaneous injection of liraglutide (25 nmol/kg, once daily) for 8 weeks | 1. WT 2. 5×FAD 3. 5×FAD+liraglutide | Morris water maze tests & nissl staining & post-synaptic density & synaptic cleft width & synaptic interface curvature & measurements of intracellular and mitochondrial ROS & ATP assay & levels of PSD95, SYN, HIF-1α, Hexokinase Ⅰ, Hexokinase Ⅱ, PKM 1/2, PKM 2, PFKFB3, LDHA, PDK 2, p-PDH, t-PDH, EAAT 1 and EAAT2 & measurements of oxygen consumption rate & lactate assay & NDA+ and NDAH measurements & glutamate uptake assay | Liraglutide improved spatial cognition and altered brain proteomic profiles in 5×FAD mice.  Liraglutide improved aerobic glycolysis and alleviated oxidative stress in the cortices of 5×FAD mice.  GLP-1 improved supportive role of astrocytes to neurons by elevating aerobic glycolysis. |
| Lixisenatide | McClean et al. (2014) [157] | APP_swe_/PS1_ΔE9_ mice/7 months | A mouse model of AD | Lixisenatide 10nmol/kg or 1 nmol/kg  Liraglutide 25nm/kg or 2.5 nmol/kg  i.p. once daily for 10 weeks | 1. WT+Saline 2. WT+Lixisenatide-1 3. WT+Liraglutide-2.5 4. WT+Lixisenatide-10 5. WT+Liraglutide-25 6. APP/PS1+Saline 7. APP/PS1+Lixisenatide-1 8. APP/PS1+Liraglutide-2.5 9. APP/PS1+Lixisenatide-10 10. APP/PS1+Liraglutide-25 | Object recognition task & LTP recording in hippocampus area CA1 & histologial staining for Iba-1, Aβ plaques, congophilic dense-core amyloid plaques, synaptophysin and doublecortin | Both drugs at all dose tested improved performance in object recognition task in APP/PS1 mice.  Both drugs increased LTP in APP/PS1 mice while lixisenatide 1 nmol/kg was most effective.  Both drugs prevented reduction of synapse numbers in APP/PS1 mice.  Both drugs at all doses reduced amyloid plaque load and dense-core Congo red positive plaque load in cortex.  All treatments reduced chronic inflammation response (microglia activation). |
| Lixisenatide | Cai et al. (2014) [179] | Male Sprague-Dawley (SD) rats/200-230 g | Aβ_25-35_ 5 nmol/μl twice injected into bilateral hippocampi | Lixisenatide 5 nmol/μl twice injected into bilateral hippocampi | 1. Vehicle+Saline 2. Vehicle+Aβ_25-35_ 3. Lixisenatide+Saline 4. Lixisenatide+ Aβ_25-35_ | Morris water maze task & in vivo hippocampal LTP recording & GSK3β and pGSK3β levels | Lixisenatide prevented Aβ_25-35_-induced impairments in spatial learning and memory.  Lixisenatide protected against Aβ_25-35_-induced suppression of hippocampal LTP.  Lixisenatide suppressed Aβ_25-35_-induced activation of GSK3β in hippocampus. |
| Lixisenatide | Cai et al. (2017) [206] | Male Sprague-Dawley (SD) rats/200-230 g | Aβ_25-35_ 5 nmol/μl bilaterally injected into hippocampi | Lixisenatide 5 nmol/μl bilaterally injected into hippocampi 15 min before Aβ_25-35_ | 1. Vehicle+Saline 2. Vehicle+Aβ_25-35_ 3. Lixisenatide+Saline 4. Lixisenatide+ Aβ_25-35_ | Y maze test & cell viability assay & pAkt and p-MERK 1/2 (Ser217/221) levels & confocal calcium imaging | Lixisenatide reversed Aβ_25-35_-induced impairments in spatial working memory.  Lixisenatide inhibited Aβ_25-35_-induced cytotoxicity on cultured hippocampal cells.  Lixisenatide relieved Aβ_25-35_-induced suppression of Akt-MEK 1/2 signaling pathway.  Lixisenatide significantly protected against Aβ_25-35_-induced elevation of [Ca^2+^]_i_. |
| Lixisenatide | Cai et al. (2018) [144] | Female APP/PS1/tau mice | Triple amyloid precursor protein/presenilin-1/tau protein AD transgenic mice | Lixisenatide 10 nmol/kg i.p. once daily for 60 days | 1. WT+Saline 2. APP/PS1/tau+Saline 3. WT+Lixisenatide 4. APP/PS1/tau+Lixisenatide | Aβ, tau and Iba-1 immunohistochemical staining & p-PKA, PKA, p-CREB, CREB, p-p38 and p38 levels | Lixisenatide reduced amyloid plaque load, neurofibrillary tangles and neuroinflammation in hippocampi of APP/PS1/tau mice  Lixisenatide relieved suppression of PKA-CREB signaling pathway, and inhibited activation of p38-MAPK in APP/PS1/tau mice. |
| Dulaglutide | Zhou et al. (2019) [150] | Male C57/BL6 mice | STZ 3 mg/kg injection into bilateral i.c.v. | Dulaglutide 0.6 mg/kg/week i.p. and exendin (9-39) 0.67 mg/kg/week | 1. Control 2. STZ 3. STZ+Dulaglutide 4. STZ+Dulaglutide+exendin (9-39) | Morris water maze test & phosphorylation of NF detection & p-tau, tau, SMI31, R61d, GLP-1, GLP-1R, pPI3K, PI3K, pAKT, AKT, pGSK3β and GSK3β levels | Dulaglutide improved learning and memory impairment of STZ mice.  Dulaglutide reduced STZ-induced phosphorylation levels of tau and NFs.  Dulaglutide improved PI3K/AKT/GSK3β pathway in STZ mice brain. |
| Exenatide | Bomba et al. (2013) [207] | 3×Tg-AD and PS1-KI mice/3 months | Models of neuronal dysfunction: (1) The presenilin-1 knock in (PS1-KI) mice. (2) Triple transgenic (PS1_M146V_, APP_swe_, tau_P301L_) mouse model of AD (3×Tg-AD) | Exenatide 500 μg/kg i.p. for 9 months | 1. PS-1-KI+Exenatide 2. PS1-KI+Saline 3. 3×Tg-AD+Exenatide 4. 3×Tg-AD+Saline | Morris water maze & p-tau and Aβ pathology & cytochrome oxidase activity measurement & analysis of cytosolic LDH activities & GC-MS analysis of brain tissue | Exenatide improved short- and long-term memory as well as brain forward LDH activity and anaerobic glucose catabolism in PS-KI mice, but had no effects on 3×Tg-AD mice.  Exenatide did no counteract age-dependent development of Aβ and tau pathway in 3×Tg-AD mice, and did not affect brain mitochondrial COX activity in two transgenic mice. |
| Exenatide | Solmaz et al. (2015) [140] | Male Sprague Dawley albino mice/200-220 g | STZ 3mg/kg i.c.v. | Exenatide 20 μg/kg/d i.p. for two weeks | 1. Sham 2. STZ+Saline 3. STZ+Exenatide | Passive avoidance learning task & histopathological evaluation & detection of brain TNF-α levels & determination of brain choline acetyl transferase (ChAT) activity & hippocampal neuronal count | Exenatide suppressed increase TNF-α levels, and significantly preserved brain ChAT activity.  Exenatide improved memory of mice, and significantly prevented decrease in hippocampal neurons. |
| Exenatide | Bomba et al. (2018) [203] | Mice/10 months | Adult mice at 10 months of age comparable to mild-life stage of humans | Exenatide 500 μg/kg i.p. 5 days per week | 1. Control 2. Exenatide | Morris water maze & cytochrome c oxidase activity & cytosolic lactate dehydrogenase activity & dendritic spine imaging & p-CREB, BDNF, TrkB, p75NTR, Perk_1,2_, PSD95, pERK5, pJNK and ERK5 levels | Exenatide improved LTM performance and promoted BDNF signaling in mice.  Exenatide reduced p75NTR signaling in mice.  Exenatide increased neuronal spine density *in vitro*. |
| Exenatide | An et al. (2019) [84] | Male 5×FAD mice/5 months | Expression APP K670 N/M671 L+I716 V+V717 I and PS1 M146 L+L286 V under the control of neuron-specific Thy-1 promoter in 5×FAD mice, resulting in overproduction of Aβ | Exenatide 100 μg/kg subcutaneous injection twice daily for 16 weeks | 1. Control 2. Control+Exenatide 3. 5×FAD 4. 5×FAD+Exenatide | Morris water maze test & synapses and mitochondria analysis through transmission electron microscopy & Aβ_1-42_ levels detection via immunohistochemistry & oxidative stress assessment & ATP content and activity of respiratory chain complex Ⅰ& levels of PSD95, SYN, Opa1 and Mfn2 | Exenatide improved learning and spatial memory ability of 5×FAD mice.  Exenatide reduced Aβ_1-42_ deposition and synaptic degradation in hippocampal CA1 region of 5×FAD mice.  Exenatide improved mitochondrial morphology and dynamics, and alleviated oxidative stress as well as energy crisis in hippocampus of 5×FAD mice. |
| Exenatide | Bomba et al. (2019) [141] | 3×Tg-AD mice/6 months | A widely-employed preclinical model of AD harboring three mutations associated with familial AD (APP Swedish, MAPT P301L, and PSEN1 M146V) and offers unique advantage of combining both Aβ- and tau-related pathology | Exenatide 500 μg/kg i.p. 5 days per week for 3 months | 1. 3×Tg-AD^CD^+Vehicel 2. 3×Tg-AD^HFD^+Vehicle 3. 3×Tg-AD^CD^+Exenatide 4. 3×Tg-AD^HFD^+Exenatide | Insulin sensitivity and glucose tolerance tests & plasma insulin assay and HOMA-IR assessment & Aβ and p-tau immunohistochemistry & morris water maze test & object recognition test & BDNF, pCREB, CREB, pTrkB, TrkB, pERK5, ERK5, pSyn, Syn, PSD95 and Pirs1 levels | Exenatide positively affected BDNF signaling in 3×Tg-AD^CD^ and prevented development of neurotrophic signaling impairment in 3×Tg-AD^HFD^ mice.  Exenatide decreased p75NTR activation in 3×Tg-AD^CD^ mice and prevented neurotoxic signaling in 3×Tg-AD^HFD^ mice. |
| Exenatide | Robinson et al. (2019) [213] | Male Tg2576 mice/4 months | Expression human APP with “Swedish” mutation | Intranasal treatment insulin 0.43×10^-3^ IU+5 μg bovine serum albumin (BSA) and exenatide 0.075 μg+5μg BSA | 1. WT+Saline 2. Tg+Saline 3. Tg+Insulin 4. Tg+Exenatide 5. Tg+Insulin+Exenatide | Insulin and glucose tolerance test & blood insulin & morris water maze & insoluble Aβ_1-42_ levels in hippocampus | Combination of insulin and exenatide lowered IRSP expressions.  Exenatide significant improved spatial learning in Morris water maze task. |
| Exenatide | Zhang et al. (2022) [148] | Male 5×FAD mice/5 months | Overexpression mutations in amyloid precursor protein gene (Swe^K670N, M671L^, Lon^V717I^, and Flo^I716V^) and in presenilin 1 gene (M146L and L286V) under Thy1 promoter | Subcutaneous injection of exenatide twice daily at 100 μg/kg per dose for 16 weeks | 1. WT 2. 5×FAD 3. 5×FAD+exenatide | Morris water maze test & Aβ_1-42_, GFAP and NLRP2 levels in piriform cortex & MTT assaying & ROS detection & IL-1β and TNF-α levels in homogenized cortical tissues & concentrations of ROS, IL-1β, TNF-α and IL-18 in supernatants of astrocyte cultures | Exenatide reduced Aβ_1-42_ deposition, expression of GFAP and NLRP2, inflammatory response, and neurodegeneration in piriform cortex of 5×FAD mice.  Exenatide reduced expression of GFAP and NLRP2, and attenuated Aβ_1-42_-induced oxidative stress and inflammatory factors, and lowered mRNA levels of NLRP2 and caspase-1 in astrocytes *in vitro*.  Exenatide reduced expression of NLRP2 inflammasome-modulated signaling pathway components, and reduced oxidative stress and inflammatory factors levels in primary astrocytes overexpressing NLRP2. |
| GLP-1 | Zhang et al. (2021) [194] | Male Wistar rats/6 weeks | A single dose intraperitoneal 60 mg/kg STZ | 0 nM, 50 nM, 100 nM, 200 nM, 500 nM GLP-1 concentration in medium with PC12 cells | 1. Control rat 2. Diabetic rat | Morris water maze & TUNEL assay & levels of Bcl2, Bax, GLP-1R and PPAR-γ & plasmatic concentration of GLP-1 and CML measurements | GLP-1 restored CML-induced apoptosis and upregulated PPAR-γ levels in PC12 cells.  Inhibition of PPAR-γ abolished protective effect of GLP-1 and impaired expression of GLP-1R.  CML concentration increased and GLP-1 concentration decreased in diabetic rats, and more cells apoptosis as well as decreased GLP-1R and PPAR-γ in brain. |
| DA-JC4 (dual GLP-1/GIP receptor agonist) | Shi et al. (2017) [233] | Male Sprague-Dawley rats/210-230 g | STZ 3 mg/kg i.c.v. | DA-JC4 10 nmol/kg i.p. daily for 2 weeks | 1. Control 2. DA-JC4 3. STZ 4. STZ+DA-JC4 | Y-maze test & morris water maze test & p-tau levels in cortex and hippocampus & IRS-1, p-IRS-1, Akt, p-Akt, Bcl-2 and Bax levels in cortex and hippocampus | DA-JC4 improved STZ-induced learning and memory impairment.  DA-JC4 reduced levels of phosphorylated tau protein.  DA-JC4 attenuated STZ-induced chronic inflammation response and apoptotic signaling.  DA-JC4 promoted re-sensitization of insulin signaling in brain. |
| DA-JC4 (dual GLP-1/GIP receptor agonist) | Maskery et al. (2020) [234] | APP/PS1 mice/9 months | APP_swe_/PS1_ΔE9_ transgenic mice | DA-JC4 0.1, 1, 10 nmol/kg i.p. injection;  Liraglutide or DA-JC4 10 nmol/kg i.p. once-daily for 8 weeks | 1. WT+Saline 2. APP/PS1+Saline 3. APP/PS1+DA-JC4-0.1 4. APP/PS1+DA-JC4-1 5. APP/PS1+DA-JC4-10 6. APP/PS1+Liraglutide-10 | Water maze test & LTP recordings in hippocampus area CA1 & immunohistochemical staining for GFAP, IBA 1 and Aβ & IL-1β and TNF-α levels | DA-JC4 dose-dependently reduced inflammation response and amyloid plaques in brain of APP/PS1 mice.  DA-JC4 was more effective than Liraglutide in reversing memory loss, enhancing synaptic plasticity in hippocampus, reducing amyloid plaques and lowering pro-inflammatory cytokine levels in brain. |
| DA-JC4 (dual GLP-1/GIP receptor agonist) | Cai et al. (2021) [235] | 3×Tg-AD mice/9 months | APP/PS1/tau transgenic mice | DA-JC4 10 nmol/kg i.p. daily for consecutive 46 days | 1. WT+PBS 2. WT+DA-JC4 3. 3×Tg-AD+PBS 4. 3×Tg-AD+DA-JC4 | Open field test & new object recognition test & Y maze test & morris water maze task & conditional fear memory test & in vivo LTP recording & PSD95, SYP, PINK1, Parkin and P62 levels & observation of synapse and mitochondria through transmission electron microscope (TEM) & dendritic spines calculation & Aβ and tau pathology in hippocampus | DA-JC4 promoted ability of 3×Tg-AD to recognize new object.  DA-JC4 improved spatial working memory, and reversed impairment of spatial reference memory as well as cognitive flexibility of 3×Tg-AD mice.  DA-JC4 enhanced ability of 3×Tg-AD mice to remember fear.  DA-JC4 improved hippocampal synaptic plasticity of 3×Tg-AD mice.  DA-JC4 increased levels of PSD95 and SYP, and alleviated hippocampal Aβ and tau pathology in 3×Tg-AD mice.  DA-JC4 normalized synapse, dendritic spines and mitochondrial numbers of 3×Tg-AD mice. |
| DA-JC1 (GLP-1/GIP dual agonist) | Salles et al. (2020) [186] | APP/PS1 mice | APPswe/PS1 dE9 transgenic mice | DA-JC1 50 nmol/kg or Liraglutide 25 nmol/kg i.p. daily for 4 weeks | 1. WT+Saline 2. WT+Liraglutide 3. WT+DA-JC1 4. APP/PS1+Saline 5. APP/PS1+Liraglutide 6. APP/PS1+DA-JC1 | Cell viability & mitochondrial ROS detection & genotoxicity & immunohistochemical staining for DCX, GFAP, Iba-1 and Aβ | DA-JC1 protected and enhanced SH-SY5Y cells viability against oxidative stress more than liraglutide and both peptides prevented ROS production and DNA damage induced by H_2_O_2_.  DA-JC1 and liraglutide equally improved neurogenesis, and attenuated Aβ oligomers in APP/PS1 mice.  DA-JC1 decreased neuroinflammatory markers more than liraglutide in hippocampus of aged and impaired mice brain. |
| DA5-CH (dual GLP-1/GIP receptor agonist) | Cao et al. (2018) [236] | APP/PS1 mice/9 months | APPswe/PS1 dE9 transgenic mice | DA5-CH 10 nmol/kg i.p. daily for 28 days | 1. WT+Saline 2. WT+DA5-CH 3. APP/PS1+Saline 4. APP/PS1+DA5-CH | Open field test & Y-maze test & spatial water maze and reversal water maze test & in vivo hippocampal L-LTP recording & immunofluorescence of Aβ plaques and p-tau protein in hippocampus & p-PI3K, t-PI3K, p-AKT, t-AKT, p-GSK3β and t-GSK3β levels | DA5-CH improved working memory of APP/PS1 mice in Y-maze test.  DA5-CH ameliorated spatial cognition deficits, and protected relearning ability as well as cognitive flexibility of APP/PS1 mice in morris water maze test.  DA5-CH effectively reversed impairment of hippocampal synaptic plasticity, and reduced hippocampal Aβ plaque load as well as tau phosphorylation in APP/PS1 mice.  DA5-CH normalized PI3K/AKT/GSK3β signaling activity in hippocampus of APP/PS1 mice. |
| DA5-CH (dual GLP-1/GIP receptor agonist) | Li et al. (2020) [237] | Male Sprague Dawley rats/250-300 g | Streptozotocin (STZ) 3 mg/kg for lateral ventricular injection | DA5-CH i.p. injection at a dose of 10 nmol/kg for 14 consecutive days | 1. Control 2. DA5-CH 3. STZ 4. STZ+DA5-CH | Blood-brain barrier penetration study & Y-maze test & morris water maze test & in vivo recording of local field potentials in hippocampal CA1 region & immunohistochemical staining for p-tau, SYN and PSD95 & levels of P-tau, t-tau, SYN, PSD95, Bax, Bcl-2, P-CREB and CREB | DA5-CH showed best BBB penetration.  DA5-CH ameliorated working memory, and reversed memory impairment in STZ-treated rats.  DA5-CH reduced hippocampal tau^S396^ phosphorylation, and significantly increased theta band energy in hippocampal area CA1.  DA5-CH reversed synaptic protein levels in hippocampus of rat brains.  DA5-CH rescued increase of Bax/Bcl-2 ratio and decrease of p-CREB^S133^ in hippocampus induced by STZ. |
| DA-CH3 (dual GLP-1/GIP receptor agonist) | Panagaki et al. (2018) [238] | Male APP_SWE_/PS1_ΔE9_ mice/10 months | A transgenic mouse model of AD | DA-CH3 i.p. once daily at a dose of 10 ml/kg/day or 25 nmol/kg/day for 8 weeks | 1. WT+Saline 2. APP_SWE_/PS1_ΔE9_+Saline 3. APP_SWE_/PS1_ΔE9_+DA-CH3 | Open field test & morris water maze task & visible-platform training & spatial acquisition & spatial reversal & immunohistochemical staining for Aβ plaques, IBA-1 and GFAP & levels of Sys, PSD95, BiP, Chop, CASP12, Beclin-1, Atg3, Atg7, LC3, Akt, GSK3β and ERK1/2 | DA-CH3 did not affect body weight of APP_SWE_/PS1_ΔE9_ mice.  DA-CH3 ameliorated aberrant exploratory locomotion, and reversed spatial learning and impairments of the APP_SWE_/PS1_ΔE9_ mice.  DA-CH3 ameliorated AD-related pathognomonic features (Aβ and inflammation), and preserved synapse integrity in APP_SWE_/PS1_ΔE9_ mice.  DA-CH3 alleviated endoplasmic reticulum (ER) stress and autophagy impairments, and restored Akt signaling in APP_SWE_/PS1_ΔE9_ mice. |
| Triple GLP-1/GIP/glucagon receptor agonist (TA) | Tai et al. (2018) [239] | APP/PS1 mice/6 months | A transgenic mouse model of AD | TA 10 nmol/kg i.p. once-daily injection for two months | 1. Control 2. APP/PS1 3. APP/PS1+TA | Morris water maze test & immunohistochemical staining for DCX, GFAP, IBA-1, 4-HNE and 8-OHdG & levels of SYN, Bcl-2, BAX, BDNF | TA improved learning and memory impairment of APP/PS1 mice.  TA ameliorated amyloid plaques, inflammation response and oxidative stress in brains of APP/PS1 mice.  TA increased immature neurons in dentate gyrus of APP/PS1 mice.  TA reversed decrease of BDNF and synaptophysin levels in hippocampus of APP/PS1 mice.  TA showed potential anti-apoptotic effects by decreasing BAX/Bcl-2 ratio in APP/PS1 mice. |
| Triagonist (GLP-1/GIP/Gcg receptor triagonist) | Li et al. (2018) [240] | 3×Tg-AD mice/7 months | 3×Tg-AD mice harboring human gene mutations in APP_Swe_, tau_P30IL_, and PS1_M146V_ | Triagonist 10 nmol/kg i.p. daily for 30 days | 1. WT+Saline 2. WT+Triagonist 3. 3×Tg+Saline 4. 3×Tg+Triagonist | Determination of blood glucose levels & open field test & Y maze test & morris water maze test & in vivo LTP recording & Aβ plaque load and tau levels analysis via histology & levels of ^S133^p-CREB, CREB, ^T286^p-CAMKⅡ, CAMKⅡ, ^S9^p-GSK3β and GSK3β | GLP-1/GIP/Gcg triagonist reduced behavioral disinhibition and improved working memory as well as long-term spatial memory of 3×Tg-AD mice.  GLP-1/GIP/Gcg triagonist improved hippocampal synaptic plasticity, and alleviated hippocampal Aβ and tau pathology in 3×Tg-AD mice.  GLP-1/GIP/Gcg triagonist upregulated expression levels of ^S133^p-CREB, ^T286^p-CAMKⅡ and ^S9^p-GSK3β in hippocampus of 3×Tg-AD mice. |
| Triagonist (GLP-1/GIP/Gcg receptor triagonist) | Li et al. (2020) [241] | 3×Tg-AD mice/7 months | 3×Tg-AD mice overexpressing human APP_Swe_, human tau_P30IL_, and PS1_M146V_ | Triagonist 10 nmol/kg i.p. daily for consecutive days and during 15 days of radial arm maze task | 1. WT+Saline 2. WT+Triagonist 3. 3×Tg+Saline 4. 3×Tg+Triagonist | Radial arm maze task & whole-cell patch clamp recording & transmembrane Ca^2+^ flux measurements & Synaptophysin and PSD-95 levels | Triagonist alleviated memory impairment in 3×Tg-AD mice in radial arm maze task.  Triagonist enhanced spontaneous excitatory synaptic activities, and mitigated abnormal hyperexcitability of hippocampal neurons in 3×Tg-AD mice.  Triagonist normalized transmembrane Ca^2+^ fluxes, and upregulated expression levels of synaptophysin as well as PSD-95 in hippocampus in 3×Tg-AD mice. |
| Oxyntomodulin (dual GLP-1 and Gcg receptor agonist) | Wang et al. (2020) [242] | Male APP/PS1 mice/2 months | APPswe/PS1 dE9 transgenic mice | Oxyntomodulin (Oxm) 25 nmol/kg i.p. daily for 2 weeks and during behavioral experiments | 1. WT+Saline 2. WT+(D-Ser2) Oxm 3. APP/PS1+Saline 4. APP/PS1+(D-Ser2) Oxm | Y maze test & morris water maze task & in vivo hippocampal LTP recording & Aβ levels in hippocampus & p-PI3K, p-AKT1, p-GSK3β levels | (D-Ser2) Oxm prevented spatial working memory deficits of APP/PS1 mice in Y maze test and Morris water maze task.  (D-Ser2) Oxm reversed LTP impairments, and reduced amyloid plaques number in hippocampal CA1 area in PP/PS1 mice.  (D-Ser2) Oxm normalized PI3K/AKT1/GSK3β signaling pathway. |

26. Abd El-Rady NM, Ahmed A, Abdel-Rady MM, Ismail OI. Glucagon-like peptide-1 analog improves neuronal and behavioral impairment and promotes neuroprotection in a rat model of aluminum-induced dementia. Physiol Rep (2021) 8(24):e14651.doi: 10.14814/phy2.14651.

28. An FM, Chen S, Xu Z, Yin L, Wang Y, Liu AR, et al. Glucagon-like peptide-1 regulates mitochondrial biogenesis and tau phosphorylation against advanced glycation end product-induced neuronal insult: Studies in vivo and in vitro. Neuroscience (2015) 300:75–84. doi: 10.1016/j.neuroscience.2015.05.023

84. An J, Zhou Y, Zhang M, Xie Y, Ke S, Liu L, et al. Exenatide alleviates mitochondrial dysfunction and cognitive impairment in the 5xFAD mouse model of alzheimer’s disease. Behav Brain Res (2019) 370:111932. doi: 10.1016/ j.bbr.2019.111932

85. Wang X, Wang L, Jiang R, Xu Y, Zhao X, Li Y. Exendin-4 antagonizes Abeta1-42-induced attenuation of spatial learning and memory ability. Exp Ther Med (2016) 12(5):2885–92. doi: 10.3892/etm.2016.3742

140. Solmaz V, Cinar BP, Yigitturk G, Cavusoglu T, Taskiran D, Erbas O. Exenatide reduces TNF-alpha expression and improves hippocampal neuron numbers and memory in streptozotocin treated rats. Eur J Pharmacol (2015) 765:482–7. doi: 10.1016/j.ejphar.2015.09.024

141. Bomba M, Granzotto A, Castelli V, Onofrj M, Lattanzio R, Cimini A, et al. Exenatide reverts the high-Fat-Diet-Induced impairment of BDNF signaling and inflammatory response in an animal model of alzheimer’s disease. J Alzheimers Dis (2019) 70(3):793–810. doi: 10.3233/JAD-190237

143. McClean PL, Jalewa J, Holscher C. Prophylactic liraglutide treatment prevents amyloid plaque deposition, chronic inflammation and memory impairment in APP/PS1 mice. Behav Brain Res (2015) 293:96–106. doi: 10.1016/ j.bbr.2015.07.024

144. Cai HY, Yang JT, Wang ZJ, Zhang J, Yang W, Wu MN, et al. Lixisenatide reduces amyloid plaques, neurofibrillary tangles and neuroinflammation in an APP/PS1/tau mouse model of alzheimer’s disease. Biochem Biophys Res Commun (2018) 495(1):1034–40. doi: 10.1016/j.bbrc.2017.11.114

146. Duarte AI, Candeias E, Alves IN, Mena D, Silva DF, Machado NJ, et al. Liraglutide protects against brain amyloid-beta1-42 accumulation in female mice with early alzheimer’s disease-like pathology by partially rescuing Oxidative/ Nitrosative stress and inflammation. Int J Mol Sci (2020) 21(5):1746. doi: 10.3390/ ijms21051746

147. Paladugu L, Gharaibeh A, Kolli N, Learman C, Hall TC, Li L, et al. Liraglutide has anti-inflammatory and anti-amyloid properties in streptozotocininduced and 5xFAD mouse models of alzheimer’s disease. Int J Mol Sci (2021) 22 (2):860. doi: 10.3390/ijms22020860

148. Zhang M, Wu Y, Gao R, Chen X, Chen R, Chen Z. Glucagon-like peptide-1 analogs mitigate neuroinflammation in alzheimer’s disease by suppressing NLRP2 activation in astrocytes. Mol Cell Endocrinol (2022) 542:111529. doi: 10.1016/ j.mce.2021.111529

149. Li L, Zhang ZF, Holscher C, Gao C, Jiang YH, Liu YZ. (Val(8)) glucagonlike peptide-1 prevents tau hyperphosphorylation, impairment of spatial learning and ultra-structural cellular damage induced by streptozotocin in rat brains. Eur J Pharmacol (2012) 674(2-3):280–6. doi: 10.1016/j.ejphar.2011.11.005

150. Zhou M, Chen S, Peng P, Gu Z, Yu J, Zhao G, et al. Dulaglutide ameliorates STZ induced AD-like impairment of learning and memory ability by modulating hyperphosphorylation of tau and NFs through GSK3beta. Biochem Biophys Res Commun (2019) 511(1):154–60. doi: 10.1016/j.bbrc.2019.01.103

151. Li Y, Duffy KB, Ottinger MA, Ray B, Bailey JA, Holloway HW, et al. GLP-1 receptor stimulation reduces amyloid-b peptide accumulation and cytotoxicity in cellular and animal models of alzheimer’s disease. J Alzheimer’s Dis (2010) 19 (4):1205–19. doi: 10.3233/JAD-2010-1314

152. Perry T, Lahiri DK, Sambamurti K, Chen D, Mattson MP, Egan JM, et al. Glucagon-like peptide-1 decreases endogenous amyloid-beta peptide (Abeta) levels and protects hippocampal neurons from death induced by abeta and iron. J Neurosci Res (2003) 72(5):603–12. doi: 10.1002/jnr.10611

154. Yang Y, Ma D, Xu W, Chen F, Du T, Yue W, et al. Exendin-4 reduces tau hyperphosphorylation in type 2 diabetic rats via increasing brain insulin level. Mol Cell Neurosci (2016) 70:68–75. doi: 10.1016/j.mcn.2015.10.005

155. Xu W, Yang Y, Yuan G, Zhu W, Ma D, Hu S. Exendin-4, a glucagon-like peptide-1 receptor agonist, reduces Alzheimer disease-associated tau hyperphosphorylation in the hippocampus of rats with type 2 diabetes. J Investig Med (2015) 63(2):267–72. doi: 10.1097/JIM.0000000000000129

156. King MR, Anderson NJ, Deciu M, Guernsey LS, Cundiff M, Hajizadeh S, et al. Insulin deficiency, but not resistance, exaggerates cognitive deficits in transgenic mice expressing human amyloid and tau proteins. reversal by exendin-4 treatment. J Neurosci Res (2020) 98(11):2357–69. doi: 10.1002/jnr.24706

157. McClean PL, Holscher C. Lixisenatide, a drug developed to treat type 2 diabetes, shows neuroprotective effects in a mouse model of alzheimer’s disease. Neuropharmacology (2014) 86:241–58. doi: 10.1016/j.neuropharm.2014.07.015

158. McClean PL, Hölscher C. Liraglutide can reverse memory impairment, synaptic loss and reduce plaque load in aged APP/PS1 mice, a model of alzheimer’s disease. Neuropharmacology (2014) 76:57 – 67. doi: 10.1016/ j.neuropharm.2013.08.005

159. Holubova M, Hruba L, Popelova A, Bencze M, Prazienkova V, Gengler S, et al. Liraglutide and a lipidized analog of prolactin-releasing peptide show neuroprotective effects in a mouse model of beta-amyloid pathology. Neuropharmacology (2019) 144:377–87. doi: 10.1016/j.neuropharm.2018.11.002

161. Ma DL, Chen FQ, Xu WJ, Yue WZ, Yuan G, Yang Y. Early intervention with glucagon-like peptide 1 analog liraglutide prevents tau hyperphosphorylation in diabetic db/db mice. J Neurochem (2015) 135(2):301–8. doi: 10.1111/jnc.13248

162. Zhang Y, Xie JZ, Xu XY, Hu J, Xu T, Jin S, et al. Liraglutide ameliorates hyperhomocysteinemia-induced Alzheimer-like pathology and memory deficits in rats via multi-molecular targeting. Neurosci Bull (2019) 35(4):724–34. doi: 10.1007/ s12264-018-00336-7

163. Hansen HH, Barkholt P, Fabricius K, Jelsing J, Terwel D, Pyke C, et al. The GLP-1 receptor agonist liraglutide reduces pathology-specific tau phosphorylation and improves motor function in a transgenic hTauP301L mouse model of tauopathy. Brain Res (2016) 1634:158–70. doi: 10.1016/j.brainres.2015.12.052

164. Qi L, Ke L, Liu X, Liao L, Ke S, Liu X, et al. Subcutaneous administration of liraglutide ameliorates learning and memory impairment by modulating tau hyperphosphorylation via the glycogen synthase kinase-3beta pathway in an amyloid beta protein induced alzheimer disease mouse model. Eur J Pharmacol (2016) 783:23–32. doi: 10.1016/j.ejphar.2016.04.052

168. Parthsarathy V, Holscher C. Chronic treatment with the GLP1 analogue liraglutide increases cell proliferation and differentiation into neurons in an AD mouse model. PloS One (2013) 8(3):e58784. doi: 10.1371/journal.pone.0058784

169. Hansen HH, Fabricius K, Barkholt P, Niehoff ML, Morley JE, Jelsing J, et al. The GLP-1 receptor agonist liraglutide improves memory function and increases hippocampal CA1 neuronal numbers in a senescence-accelerated mouse model of alzheimer’s disease. J Alzheimers Dis (2015) 46(4):877–88. doi: 10.3233/JAD-143090

171. Gault VA, Holscher C. GLP-1 agonists facilitate hippocampal LTP and reverse the impairment of LTP induced by beta-amyloid. Eur J Pharmacol (2008) 587(1-3):112–7. doi: 10.1016/j.ejphar.2008.03.025

172. Gengler S, McClean PL, McCurtin R, Gault VA, Holscher C. Val(8)GLP-1 rescues synaptic plasticity and reduces dense core plaques in APP/PS1 mice. Neurobiol Aging (2012) 33(2):265–76. doi: 10.1016/j.neurobiolaging.2010.02.014

173. Wang XH, Yang W, Holscher C, Wang ZJ, Cai HY, Li QS, et al. Val(8)- GLP-1 remodels synaptic activity and intracellular calcium homeostasis impaired by amyloid beta peptide in rats. J Neurosci Res (2013) 91(4):568–77. doi: 10.1002/ jnr.23181

175. McClean PL, Parthsarathy V, Faivre E, Holscher C. The diabetes drug liraglutide prevents degenerative processes in a mouse model of alzheimer’s disease. J Neurosci (2011) 31(17):6587–94. doi: 10.1523/JNEUROSCI.0529-11.2011

176. Batista AF, Forny-Germano L, Clarke JR, Lyra E Silva NM, Brito-Moreira J, Boehnke SE, et al. The diabetes drug liraglutide reverses cognitive impairment in mice and attenuates insulin receptor and synaptic pathology in a non-human primate model of alzheimer’s disease. J Pathol (2018) 245(1):85–100. doi: 10.1002/ path.5056

177. Han WN, Holscher C, Yuan L, Yang W, Wang XH, Wu MN, et al. Liraglutide protects against amyloid-beta protein-induced impairment of spatial learning and memory in rats. Neurobiol Aging (2013) 34(2):576–88. doi: 10.1016/ j.neurobiolaging.2012.04.009

178. Ohtake N, Saito M, Eto M, Seki K. Exendin-4 promotes the membrane trafficking of the AMPA receptor GluR1 subunit and ADAM10 in the mouse neocortex. Regul Pept (2014) 190-191:1–11. doi: 10.1016/j.regpep.2014.04.003

179. Cai HY, Holscher C, Yue XH, Zhang SX, Wang XH, Qiao F, et al. Lixisenatide rescues spatial memory and synaptic plasticity from amyloid beta protein-induced impairments in rats. Neuroscience (2014) 277:6–13. doi: 10.1016/ j.neuroscience.2014.02.022

180. Zhang SX, Cai HY, Ma XW, Yuan L, Zhang J, Wang ZJ, et al. GLP-1 analogue CJC-1131 prevents amyloid b protein-induced impirments of spatial memory and synaptic plasticity in rats. Behav Brain Res (2017) 326:237–43. doi: 10.1016/j.bbr.2017.03.018

182. Chen S, Liu AR, An FM, Yao WB, Gao XD. Amelioration of neurodegenerative changes in cellular and rat models of diabetes-related alzheimer’s disease by exendin-4. Age (Dordr) (2012) 34(5):1211–24. doi: 10.1007/s11357-011-9303-8

186. Salles GN, Calio ML, Holscher C, Pacheco-Soares C, Porcionatto M, Lobo AO. Neuroprotective and restorative properties of the GLP-1/GIP dual agonist DA-JC1 compared with a GLP-1 single agonist in alzheimer’s disease. Neuropharmacology (2020) 162:107813. doi: 10.1016/j.neuropharm.2019.107813

188. Xie Y, Zheng J, Li S, Li H, Zhou Y, Zheng W, et al. GLP-1 improves the neuronal supportive ability of astrocytes in alzheimer’s disease by regulating mitochondrial dysfunction via the cAMP/PKA pathway. Biochem Pharmacol (2021) 188:114578. doi: 10.1016/j.bcp.2021.114578

189. Garabadu D, Verma J. Exendin-4 attenuates brain mitochondrial toxicity through PI3K/Akt-dependent pathway in amyloid beta (1-42)-induced cognitive deficit rats. Neurochem Int (2019) 128:39–49. doi: 10.1016/j.neuint.2019.04.006

191. Perry T, Haughey NJ, Mattson MP, Egan JM, Greig NH. Protection and reversal of excitotoxic neuronal damage by glucagon-like peptide-1 and exendin-4. J Pharmacol Exp Ther (2002) 302(3):881–8. doi: 10.1124/jpet.102.037481

194. Zhang H, Song B, Zhu W, Liu L, He X, Wang Z, et al. Glucagon-like peptide-1 attenuated carboxymethyl lysine induced neuronal apoptosis via peroxisome proliferation activated receptor-gamma. Aging (Albany NY) (2021) 13(14):19013–27. doi: 10.18632/aging.203351

203. Bomba M, Granzotto A, Castelli V, Massetti N, Silvestri E, Canzoniero LMT, et al. Exenatide exerts cognitive effects by modulating the BDNF-TrkB neurotrophic axis in adult mice. Neurobiol Aging (2018) 64:33–43. doi: 10.1016/ j.neurobiolaging.2017.12.009

205. Wang X, Wang L, Jiang R, Yuan Y, Yu Q, Li Y. Exendin-4 antagonizes Abeta1-42-induced suppression of long-term potentiation by regulating intracellular calcium homeostasis in rat hippocampal neurons. Brain Res (2015) 1627:101–8. doi: 10.1016/j.brainres.2015.09.015

206. Cai HY, Wang ZJ, Holscher C, Yuan L, Zhang J, Sun P, et al. Lixisenatide attenuates the detrimental effects of amyloid beta protein on spatial working memory and hippocampal neurons in rats. Behav Brain Res (2017) 318:28–35. doi: 10.1016/j.bbr.2016.10.033

207. Bomba M, Ciavardelli D, Silvestri E, Canzoniero LM, Lattanzio R, Chiappini P, et al. Exenatide promotes cognitive enhancement and positive brain metabolic changes in PS1-KI mice but has no effects in 3xTg-AD animals. Cell Death Dis (2013) 4:e612. doi: 10.1038/cddis.2013.139

208. Zheng J, Xie Y, Ren L, Qi L, Wu L, Pan X, et al. GLP-1 improves the supportive ability of astrocytes to neurons by promoting aerobic glycolysis in alzheimer’s disease. Mol Metab (2021) 47:101180. doi: 10.1016/ j.molmet.2021.101180

210. Carranza-Naval MJ, Del Marco A, Hierro-Bujalance C, Alves-Martinez P, Infante-Garcia C, Vargas-Soria M, et al. Liraglutide reduces vascular damage, neuronal loss, and cognitive impairment in a mixed murine model of alzheimer’s disease and type 2 diabetes. Front Aging Neurosci (2021) 13:741923. doi: 10.3389/ fnagi.2021.741923

211. Qi L, Chen Z, Wang Y, Liu X, Liu X, Ke L, et al. Subcutaneous liraglutide ameliorates methylglyoxal-induced Alzheimer-like tau pathology and cognitive impairment by modulating tau hyperphosphorylation and glycogen synthase kinase-3beta. Am J Transl Res (2017) 9(2):247–60. PMCID: PMC5340664

212. Li H, Cao L, Ren Y, Jiang Y, Xie W, Li D. GLP-1 receptor regulates cell growth through regulating IDE expression level in Abeta1-42-treated PC12 cells. Biosci Rep (2018) 38(4):BSR20171284. doi: 10.1042/BSR20171284

213. Robinson A, Lubitz I, Atrakchi-Baranes D, Licht-Murava A, Katsel P, Leroith D, et al. Combination of insulin with a GLP1 agonist is associated with better memory and normal expression of insulin receptor pathway genes in a mouse model of alzheimer’s disease. J Mol Neurosci (2019) 67(4):504–10. doi: 10.1007/s12031-019-1257-9

214. Long-Smith CM, Manning S, McClean PL, Coakley MF, O’Halloran DJ, Holscher C, et al. The diabetes drug liraglutide ameliorates aberrant insulin receptor localisation and signalling in parallel with decreasing both amyloid-beta plaque and glial pathology in a mouse model of alzheimer’s disease. Neuromolecular Med (2013) 15(1):102–14. doi: 10.1007/s12017-012-8199-5

216. Xiong H, Zheng C, Wang J, Song J, Zhao G, Shen H, et al. The neuroprotection of liraglutide on Alzheimer-like learning and memory impairment by modulating the hyperphosphorylation of tau and neurofilament proteins and insulin signaling pathways in mice. J Alzheimers Dis (2013) 37 (3):623–35. doi: 10.3233/JAD-130584

217. Wang XH, Li L, Hölscher C, Pan YF, Chen XR, Qi JS. Val8-glucagon-like peptide-1 protects against Ab1–40-induced impairment of hippocampal late-phase long-term potentiation and spatial learning in rats. Neuroscience (2010) 170 (4):1239–48. doi: 10.1016/j.neuroscience.2010.08.028

218. Ma T, Du X, Pick JE, Sui G, Brownlee M, Klann E. Glucagon-like peptide-1 cleavage product GLP-1(9-36) amide rescues synaptic plasticity and memory deficits in alzheimer’s disease model mice. J Neurosci (2012) 32(40):13701–8. doi: 10.1523/JNEUROSCI.2107-12.2012

219. Iwai T, Sawabe T, Tanimitsu K, Suzuki M, Sasaki-Hamada S, Oka J. Glucagon-like peptide-1 protects synaptic and learning functions from neuroinflammation in rodents. J Neurosci Res (2014) 92(4):446–54. doi: 10.1002/ jnr.23335

220. Gao C, Liu Y, Jiang Y, Ding J, Li L. Geniposide ameliorates learning memory deficits, reduces tau phosphorylation and decreases apoptosis via GSK3beta pathway in streptozotocin-induced alzheimer rat model. Brain Pathol (2014) 24(3):261–9. doi: 10.1111/bpa.12116

221. Lv C, Wang L, Liu X, Yan S, Yan SS, Wang Y, et al. Multi-faced neuroprotective effects of geniposide depending on the RAGE-mediated signaling in an Alzheimer mouse model. Neuropharmacology (2015) 89:175–84. doi: 10.1016/j.neuropharm.2014.09.019

222. Zhang Z, Wang X, Zhang D, Liu Y, Li L. Geniposide-mediated protection against amyloid deposition and behavioral impairment correlates with downregulation of mTOR signaling and enhanced autophagy in a mouse model of alzheimer’s disease. Aging (Albany NY) (2019) 11(2):536–48. doi: 10.18632/ aging.101759

223. Oka J, Suzuki E, Kondo Y. Endogenous GLP-1 is involved in beta-amyloid protein-induced memory impairment and hippocampal neuronal death in rats. Brain Res (2000) 878(1-2):194–8. doi: 10.1016/S0006-8993(00)02741-4

224. Bomfim TR, Forny-Germano L, Sathler LB, Brito-Moreira J, Houzel JC, Decker H, et al. An anti-diabetes agent protects the mouse brain from defective insulin signaling caused by alzheimer’s disease- associated abeta oligomers. J Clin Invest (2012) 122(4):1339–53. doi: 10.1172/JCI57256

225. Wang X, Wang L, Xu Y, Yu Q, Li L, Guo Y. Intranasal administration of exendin-4 antagonizes Abeta31-35-induced disruption of circadian rhythm and impairment of learning and memory. Aging Clin Exp Res (2016) 28(6):1259–66. doi: 10.1007/s40520-016-0548-z

226. Jia XT, Ye T, Yuan L, Zhang GJ, Liu ZQ, Di ZL, et al. Exendin-4, a glucagon-like peptide 1 receptor agonist, protects against amyloid-beta peptideinduced impairment of spatial learning and memory in rats. Physiol Behav (2016) 159:72–9. doi: 10.1016/j.physbeh.2016.03.016

227. Wang Y, Chen S, Xu Z, Chen S, Yao W, Gao X. GLP-1 receptor agonists downregulate aberrant GnT-III expression in alzheimer’s disease models through the Akt/GSK-3beta/beta-catenin signaling. Neuropharmacology (2018) 131:190–9. doi: 10.1016/j.neuropharm.2017.11.048

228. Park JS, Kam TI, Lee S, Park H, Oh Y, Kwon SH, et al. Blocking microglial activation of reactive astrocytes is neuroprotective in models of alzheimer’s disease. Acta Neuropathol Commun (2021) 9(1):78. doi: 10.1186/s40478-021-01180-z

229. Yang Y, Zhang J, Ma D, Zhang M, Hu S, Shao S, et al. Subcutaneous administration of liraglutide ameliorates Alzheimer-associated tau hyperphosphorylation in rats with type 2 diabetes. J Alzheimers Dis (2013) 37 (3):637–48. doi: 10.3233/JAD-130491

230. Spolcova A, Mikulaskova B, Holubova M, Nagelova V, Pirnik Z, Zemenova J, et al. Anorexigenic lipopeptides ameliorate central insulin signaling and attenuate tau phosphorylation in hippocampi of mice with monosodium glutamate-induced obesity. J Alzheimers Dis (2015) 45(3):823–35. doi: 10.3233/JAD-143150

231. Hansen HH, Fabricius K, Barkholt P, Kongsbak-Wismann P, Schlumberger C, Jelsing J, et al. Long-term treatment with liraglutide, a glucagon-like peptide-1 (GLP-1) receptor agonist, has no effect on beta-amyloid plaque load in two transgenic APP/PS1 mouse models of alzheimer’s disease. PloS One (2016) 11(7):e0158205. doi: 10.1371/journal.pone.0158205

232. Chen S, Sun J, Zhao G, Guo A, Chen Y, Fu R, et al. Liraglutide improves water maze learning and memory performance while reduces hyperphosphorylation of tau and neurofilaments in APP/PS1/Tau triple transgenic mice. Neurochem Res (2017) 42(8):2326–35. doi: 10.1007/s11064-017- 2250-8

233. Shi L, Zhang Z, Li L, Holscher C. A novel dual GLP-1/GIP receptor agonist alleviates cognitive decline by re-sensitizing insulin signaling in the Alzheimer icv. STZ rat model. Behav Brain Res (2017) 327:65–74. doi: 10.1016/j.bbr.2017.03.032

234. Maskery M, Goulding EM, Gengler S, Melchiorsen JU, Rosenkilde MM, Holscher C. The dual GLP-1/GIP receptor agonist DA4-JC shows superior protective properties compared to the GLP-1 analogue liraglutide in the APP/ PS1 mouse model of alzheimer’s disease. Am J Alzheimers Dis Other Demen (2020) 35:1533317520953041. doi: 10.1177/1533317520953041

235. Cai HY, Yang D, Qiao J, Yang JT, Wang ZJ, Wu MN, et al. A GLP-1/GIP dual receptor agonist DA4-JC effectively attenuates cognitive impairment and pathology in the APP/PS1/Tau model of alzheimer’s disease. J Alzheimers Dis (2021) 83(2):799–818. doi: 10.3233/JAD-210256

236. Cao Y, Hölscher C, Hu MM, Wang T, Zhao F, Bai Y, et al. DA5-CH, a novel GLP-1/GIP dual agonist, effectively ameliorates the cognitive impairments and pathology in the APP/PS1 mouse model of alzheimer’s disease. Eur J Pharmacol (2018) 827:215–26. doi: 10.1016/j.ejphar.2018.03.024

237. Li C, Liu W, Li X, Zhang Z, Qi H, Liu S, et al. The novel GLP-1/GIP analogue DA5-CH reduces tau phosphorylation and normalizes theta rhythm in the icv. STZ rat Model AD. Brain Behav (2020) 10(3):e01505. doi: 10.1002/brb3.1505

238. Panagaki T, Gengler S, Holscher C. The novel DA-CH3 dual incretin restores endoplasmic reticulum stress and autophagy impairments to attenuate Alzheimer-like pathology and cognitive decrements in the APPSWE/PS1DeltaE9 mouse model. . J Alzheimers Dis (2018) 66(1):195–218. doi: 10.3233/JAD-180584

239. Tai J, Liu W, Li Y, Li L, Holscher C. Neuroprotective effects of a triple GLP1/GIP/glucagon receptor agonist in the APP/PS1 transgenic mouse model of alzheimer’s disease. Brain Res (2018) 1678:64–74. doi: 10.1016/ j.brainres.2017.10.012

240. Li T, Jiao JJ, Holscher C, Wu MN, Zhang J, Tong JQ, et al. A novel GLP-1/ GIP/Gcg triagonist reduces cognitive deficits and pathology in the 3xTg mouse model of alzheimer’s disease. Hippocampus (2018) 28(5):358–72. doi: 10.1002/ hipo.22837

241. Li T, Jiao JJ, Su Q, Holscher C, Zhang J, Yan XD, et al. A GLP-1/GIP/Gcg receptor triagonist improves memory behavior, as well as synaptic transmission, neuronal excitability and Ca(2+) homeostasis in 3xTg-AD mice. Neuropharmacology (2020) 170:108042. doi: 10.1016/j.neuropharm.2020.108042

242. Wang ZJ, Han YF, Zhao F, Yang GZ, Yuan L, Cai HY, et al. A dual GLP-1 and gcg receptor agonist rescues spatial memory and synaptic plasticity in APP/ PS1 transgenic mice. Horm Behav (2020) 118:104640. doi: 10.1016/ j.yhbeh.2019.104640
